# Childhood environmental adversity is not linked to lower levels of cooperative behaviour in economic games

**DOI:** 10.1017/ehs.2021.21

**Published:** 2021-03-17

**Authors:** N. Lettinga, H. Mell, Y. Algan, P. O. Jacquet, C. Chevallier

**Affiliations:** 1 LNC², Département d'études cognitives, Ecole normale supérieure, Université PSL, INSERM, 75005 Paris, France; 2 Sciences Po, OFCE, 27 Rue Saint-Guillaume, 75007 Paris, France; 3 Institut Jean Nicod, Département d'études cognitives, Ecole normale supérieure, Université PSL, EHESS, CNRS, 75005 Paris, France

**Keywords:** Cooperation, life-history strategy, childhood environmental adversity, economic games

## Abstract

Cooperation is a universal phenomenon, it is present in all human cultures from hunter–gatherers to industrialised societies, and it constitutes a fundamental aspect of social relationships. There is, however, variability in the amount of resources people invest in cooperative activities. Recent findings indicate that this variability may be partly explained as a contextually appropriate response to environmental conditions. Specifically, adverse environments seem to be associated with less cooperation and recent findings suggest that this effect is partly mediated by differences in individuals’ life-history strategy. In this paper, we set out to replicate and extend these findings by measuring actual cooperative behaviour in three economic games – a Dictator game, a Trust game and a Public Goods game – on a nationally representative sample of 612 people. Although we found that the cooperation and life-history strategy latent variables were adequately captured by the models, the hypothesised relationship between childhood environmental adversity and adult cooperation and the mediation effect by life-history strategy were not found.

## Graphical abstract

**Figure:**
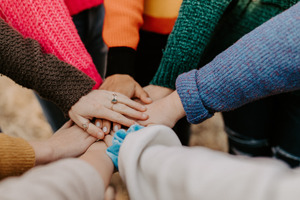

**Social media summary:** No evidence that experiencing adverse life conditions during childhood decreases cooperation later in life

## 1. Introduction

Cooperation is a universal phenomenon, it is present in all human cultures from hunter–gatherers to industrialised societies, and it constitutes a fundamental aspect of social relationships (Nowak & Highfield, 2011; Rand & Nowak, 2013). There is, however, variability in the amount of resources people invest in cooperative activities (Alesina & La Ferrara, 2002). One possible explanation for such variability is that environmental adversity has a negative impact on cooperation. People exposed to adverse environments may indeed restrict their investments, including their social investments, in what may be seen as a contextually appropriate response to adversity (Lettinga et al., 2020; McCullough et al., 2013; Wu et al., 2020).

However, current evidence linking cooperation and environmental adversity is mixed. On the one hand, when cooperation is measured via self-reported questionnaires or in field experiments, the pattern of results is rather consistent and suggestive of an association between adverse environments and decreased cooperation. In particular, a recent meta-analysis showed that early-life stress is associated with less prosocial behaviour in adulthood when it is measured via self-reports (Wu et al., 2020). Furthermore, Korndörfer et al. (2015) and Schmukle et al. (2019) found a positive correlation between social class and prosociality (e.g. more charitable, trusting and helpful) using questionnaire data from large and representative international samples (average *N* over nine studies = 20.243). Finally, wealthier households and people from wealthier neighbourhoods display more prosocial behaviours (i.e. donate more, return more lost and misdirected letters) in field experiments (Andreoni et al., 2017; Holland et al., 2012; Nettle et al., 2011; Silva & Mace, 2014, 2015).

On the other hand, when cooperation is measured using behavioural methods in the laboratory, including economic games, the results are mixed: several studies find a link between adverse environments and decreased cooperation (Korndörfer et al., 2015; McCullough et al., 2013; Nettle et al., 2011; Safra et al., 2016; Schmukle et al., 2019), but other studies find a link in the opposite direction, i.e. between adverse environments and increased cooperation (Amir et al., 2018), or no effect at all (Stamos et al., 2020, who failed to replicate Piff et al.'s (2010) initial effect; Wu et al., 2017). In a recent meta-analysis, no overall effect was found (Wu et al., 2020).

The reason why these behavioural studies produce mixed results is unclear but several explanations can be put forward. First, some of these studies have smaller sample sizes which results in lower power. In particular, this might explain why the study by Piff et al. (2010) failed to be replicated in subsequent high-powered pre-registered studies (Stamos et al., 2020). Second, they usually use one economic game per study, which may limit the generalisability of the findings (Camerer, 2011). In line with this idea, McAuliffe et al. (2019) found that a general factor based on several economic games is associated with non-game cooperation, but individual games are not. A third and more general point is that there is mounting evidence that economic games lack ecological validity (Galizzi & Navarro-Martinez, 2019). Possible explanations for this is that economic games decisions in the laboratory are performed in context-free environments with artificial rewards, choice sets and time horizons that do not extrapolate well to real-world situations (Levitt & List, 2007).

Another open question is the channel by which environmental adversity is associated with cooperation. One possible explanation is that the relationship between environmental adversity and cooperation is mediated by individuals’ life-history strategies. Life-history theory is the branch of evolutionary theory that deals with the way in which organisms allocate energy to different functions (e.g. reproduction, somatic maintenance) and with the impact of the local environment on the optimal allocation balance (Stearns, 1992). In the past decades, a growing body of experimental work demonstrated that organisms calibrate their behavioural strategies to the specific circumstances in which they live. In humans, environmental adversity appears to affect the way in which individuals deal with the reproduction–maintenance trade-off (Ellis et al., 2009; Jasienska et al., 2017; Nettle, 2010; Promislow & Harvey, 1990).

However, the evidence for life-history strategy as a mediator between environmental adversity and cooperation is also mixed. Wu et al. (2017) found that early life environment (i.e. childhood socioeconomic status and childhood unpredictability) correlated with life-history strategies (as assessed with the Mini-K scale and High-K Strategy Scale), but not with cooperation measured via economic games. In contrast, Lettinga et al. (2020) found that modulations in the reproduction–maintenance trade-off partly mediated the relationship between adversity and collective action using questionnaire data from the European Values Study and the World Values Survey. As mentioned above, an important difference between these two studies is the use of self-reports vs. economic games. These studies also use different instruments to measure life-history strategies. Wu et al. (2017) use the Mini-K scale and High-K Strategy Scale, which were recently criticised because they did not correlate with measures of mating effort (which is an important life-history trait; Olderbak et al., 2014). Similarly, the High-K Strategy Scale was recently criticised by Copping et al. (2014) for lack of construct validity. Incontrast, Lettinga et al. (2020) and our study rely on indicators which come as close as possible to testing the reproduction–maintenance trade-off (e.g. number of children, respondents’ age at their first child's birth, Ellis et al., 2009; Mell et al., 2018). One final methodological difference is that Wu et al. (2017) rely on an MTurk sample while our data was collected in a representative sample of the population, which may be more diverse in terms of both cooperation and exposure to adversity. Using these methodological improvements, together with better statistical tools, may therefore put us in a better position to test the hypothesis that differences in life-history strategies mediate the relationship between exposure to environmental adversity and cooperation.

The evolutionary rationale accounting for such mediation effects is still unclear: some authors have argued that organisms adapt their reproductive and somatic strategy to variations in extrinsic mortality (Ellis et al., 2009; Jasienska et al., 2017; Nettle, 2010; Promislow & Harvey, 1990). Environments with high mortality are indeed associated with earlier reproduction, increased number of offspring, shorter growth and diminished investment in somatic maintenance, while more favourable environments are associated with delayed reproduction, fewer offspring, longer growth and increased investment in somatic maintenance (Akee et al., 2018; Brown & Sear, 2020; Brumbach et al., 2009; Del Giudice et al., 2015; Ellis et al., 2009; Jasienska et al., 2017; Mell et al., 2018; Nettle, 2011; Promislow & Harvey, 1990; Reznick & Endler, 1982). This might occur because mortality has an effect on density-dependent competition, as demonstrated by recent modelling work in evolutionary biology (André & Rousset, 2019).

At the proximal level, researchers have also aimed to put forward psychological mechanisms accounting for the constellation of behaviours that is associated with deprivation in humans (Pepper & Nettle, 2017). One possible explanation is that adverse environments are associated with mechanisms biasing individuals towards more immediate rewards and a shorter time horizon (Bulley & Pepper, 2017; Griskevicius et al., 2011; Simpson et al., 2012; Wu et al., 2020). Such a psychological switch would have a wide impact on a range of behaviours relying on delayed gratification, including many social behaviours. Lettinga et al. (2020), for instance, have argued that cooperative variability might be guided by this longer-term calibration to environmental adversity. The reason is that cooperation is a future-oriented strategy: in the short term, it is more advantageous to reap immediate, smaller, but more certain benefits by cooperating less, but in the long term, it is more advantageous to invest in cooperation so as to reap longer-term benefits (such as increased social reputation; Axelrod & Hamilton, 1981; Baumard et al., 2013; Sjåstad, 2019; Trivers, 1971).

In the context of this ambiguous literature, the goal of this paper is twofold. The first is to address the limitations of existing studies that use economic games. Specifically, measurement error was reduced by relying on three economic games (i.e. Dictator game, Trust game, Public Goods game), performed on a single, large and representative sample. The Dictator game, the Trust game and the Public Goods game were chosen because they are among the most commonly used economic games in the literature (Amir et al., 2018; Korndörfer et al., 2015; Nettle et al., 2011; Piff et al., 2010; Safra et al., 2016; Stamos et al., 2020; Wu et al., 2017), which allowed us to compare our results more easily with the existing literature. They also cover a wide range of social preferences: altruism for the Dictator game, trust for the Trust game and cooperation for the Public Goods game (Algan et al., 2013; Levitt & List, 2007).

The second goal of our study is to replicate the mediation effect of life-history strategy between childhood environmental adversity and adults’ level of cooperation found by Lettinga et al.'s study (2020). We focus on adversity experienced early in life. The early years of life – from conception to sexual maturity – represent a sensitive period characterised by rapid development, which makes many biological systems vulnerable to environmental stressors. These stress factors, commonly referred to as environmental adversity, interfere with organisms’ developmental trajectories and can have a lasting impact on health, reproduction, cognition and behaviour (Ellis & Del Giudice, 2019). The multiple effects of early adversity on physiological and cognitive development are likely to be sequential, distributed on a continuum and ultimately obey a logic of developmental organisation. By studying the specific effect of childhood environmental adversity, we assume that adult cooperative behaviour is one of the long-term outcomes of this developmental logic, together with reproductive efforts and somatic maintenance capacity. To test this model, we applied multivariate analytic techniques (i.e. structural equation modelling) on data collected by us in collaboration with Ipsos, a French polling institute, consisting of a large sample of respondents representative of the French adult population.

Two lines of hypotheses were tested. The first line of hypotheses consists in testing (1) whether the behavioural measures extracted from the three economic games relate to a single hypothesised latent construct and (2) whether individuals’ life-history strategy – the reproduction–maintenance trade-off – can be adequately modelled as a hypothetical latent construct capturing the covariation of several indicators previously identified by Mell et al. (2018). The second line of hypotheses aims to test the association between a single composite variable thought to reflect the level of childhood adversity experienced by the respondent and the two latent variables (i.e. the reproduction–maintenance trade-off and cooperative tendencies). The two main predictions are: (3) individuals who experienced greater levels of adversity during their childhood display weaker cooperative tendencies later in life and (4) this negative association is mediated by individuals’ life-history strategy. In sum, an adverse childhood environment is associated with increased investment in reproduction and decreased investment in somatic maintenance, which in turn is associated with decreased adult cooperation.

## 2. Materials and methods

The raw data, study materials, analysis code, laboratory log and deviations from the registered report can be found at osf.io/g4scw.

### 2.1. Respondents

Our sample consisted of 612 respondents (females, *N* = 325; aged 19–83 years old; mean age = 53 ± 14 SD) and was recruited online by Ipsos in France. This strategy is suited to our purposes because exposure to adversity varies greatly between socioeconomic classes even in WEIRD (i.e., Western, Educated, Industrialized, Rich and Democratic) countries such as France. For example, average life expectancy in France for the most affluent men is 13 years higher than that of the poorest men (8 year difference for women; INSEE, 2018). Furthermore, Nettle (2015) has shown that age at first birth for women, social trust and even mean Dictator game transfer is as different between WEIRD and non-WEIRD countries as it is between a deprived and an affluent neighbourhood within a single UK city. Thus, even in a WEIRD sample, life outcomes can be substantially different between participants placed differently along the Educated and Rich gradients even within a single WEIRD country.

The respondents were collected via several steps. First, 1,691 respondents filled out a survey consisting of questions about their childhood environment and life-history strategy items (see below for a detailed description). The quota method was applied so that the end panel was representative on age, gender, geographical region, urban vs. rural and occupation. Second, 1,006 of these respondents agreed to participate in additional surveys, including the three economic games and a self-reported measure of social trust included in the study. Before running the analyses, we conducted some basic quality checks that led to the exclusion of a number of respondents: 92 respondents were removed because they gave a different answer to the question regarding gender between the first and second survey; 18 respondents were removed because they gave too many absurd answers (>3 SD from the sample mean), e.g. number of years spent smoking greater than the respondents’ age (see Table S1 of the Supplementary Materials for a full list of exclusion criteria); and 284 respondents were excluded because they reported not having children (two of the indicators used to model life-history strategy were only relevant for respondents with children). Our final sample included 612 respondents.

### 2.2. Determining sample size

Sample size requirements for structural equation models are based on many criteria (e.g. complexity of the model, distribution of the indicators, levels of missing data; Kline, 2015). However, Jackson (2003) proposed the *N*:*q* rule, which states that for every parameter one needs 20 respondents. Because our model has 51 parameters, *N* should be 1020, which is higher than the 612 participants which we have. However, based on Preacher and Coffman (2006), we calculated that the power of the proposed analysis is 0.88 (based on *N* = 612, d.f. = 40, *α* = 0.05, RMSEA.0 = 0.05, RMSEA.A = 0.07), which is higher than the generally acceptable standard of 0.8 (Cohen, 1988). Based on a similar model (Lettinga et al., 2020), we expect an effect size of 0.30 (medium effect) for the relationship between childhood environmental adversity and life-history strategy, an effect size of 0.70 (large effect) for the relationship between life-history strategy and cooperation, and an effect size of 0.10 (small effect) for the relationship between childhood environmental adversity and cooperation.

### 2.3. General procedure

The respondents first completed the survey including all childhood environmental adversity and life-history strategy items (the full questionnaire is available in Section 2 of the Supplementary Material). Respondents were contacted one week later to participate in the economic games and respond to additional surveys including a self-reported measure of social trust. The procedure for the economic games is as follows. All participants signed an informed consent form before starting the experiment on their personal computer. The experiment took place on the Lime Survey platform. The players could not communicate with each other or with the experimenter. The participants were told that they would be playing with different partners in each game. Each participant took part in three economic games: a Dictator game, a Trust game and a Public Goods game, in that order. This order was chosen to start with the easiest game, then the intermediate game and finally the hardest game in order to help participants get accustomed to the games. Each game was played once. Participants did not receive feedback about the behaviour of the ‘trustee’ in the Trust game and about the behaviour of the other players in the Public Goods game until the very end of the experiment. We did not include specific questions about whether participants understood the rules and payoffs of the games. However, at the start of each game, the instructions were presented in writing and followed by animations to clarify the game. After that, a screen was provided with examples of decisions and detailed calculations of the resulting payoffs for each player. Finally, a screen was provided where participants could practice the Trust game and the Public Goods game using an earnings calculator, to make hypothetical decisions and see the resulting outcome. Participants had the option of going back to the instruction screens during the entire duration of the experiment. Participants were told at the start of the experiment that they would be paid 14 euros for participating in the experiment plus their earnings based on the outcome of one of the economic games that was chosen randomly. Their earnings were shown at the end of the experiment. Participants received their earnings via Amazon vouchers.

### 2.4. Variables of interest

#### 2.4.1. Childhood environmental adversity

Environmental adversity consists of two different dimensions: harshness (i.e. externally caused levels of morbidity–mortality that an individual cannot control) and unpredictability (i.e. spatial–temporal variation in harshness) (Ellis et al., 2009). In a recent paper, Mell et al. (2018) put forward a questionnaire consisting of 15 items assessing both these dimensions. These indicators encompass various features of childhood environmental adversity (i.e. resource scarcity, parental investment and care, respondents’ exposure to extrinsic mortality, violence and unpredictability during childhood) and were chosen because they reflect various aspects of childhood environment that previous studies have found to be associated with one or several life-history traits in adulthood (Griskevicius et al., 2011; McCullough et al., 2013; Nettle & Cockerill, 2010; Simpson et al., 2012). Examples of questions are as follows: ‘Did your parents get divorced or separated before the age of 18?’, ‘Some of the punishments I received when I was a child now seem too harsh to me’ or ‘Did you live with one or several people who had spent time in prison?’ (see Table S2 of the Supplementary Material for the full list of items and scales).

#### 2.4.2. Life-history strategy

Life-history strategy consists of eight indicators (Mell et al., 2018) approximating the reproduction–maintenance trade-off (Ellis et al., 2009). These items have been used in the literature before; see Nettle et al. (2010) and Simpson et al. (2012) for the reproduction life-history strategy indicators and Pepper and Nettle (2014) for the somatic maintenance life-history strategy indicators. An example of a question regarding respondents’ investment in reproduction is ‘age at first child's birth’, and an example of a question regarding respondents’ investment in somatic maintenance is ‘general health status’ (see Table S3 of the Supplementary Material for the full list of items and scales).

#### 2.4.3. Cooperation

Cooperation was based on three indicators: the initial contribution in the Dictator game, the Trust game and the Public Goods game (scale 1–10).

In the Dictator game, two players were involved. Participants were always assigned to the role of the dictator. The dictator was given 10 euros and the second player was given nothing. The dictator was told that he or she could offer some amount of that money (between 0 and 10 euros) to the second player and that whatever amount the dictator offered the second player must be accepted.

In the Trust game, two players were involved. Participants were always assigned to the role of the trustor. They were matched with the response of a second player, the trustee, drawn randomly from a database of participants involved in a previous run of the same Trust game. The trustor and the trustee were each given 10 euros. The trustor was told that he or she could offer some amount of that money (between 0 and 10 euros) to the trustee. That amount was then multiplied by 3. The trustee offered some amount back (between 0 and 30 euros) to the trustor based on the previous data.

In the Public Goods game, four players were involved. The participant was matched with the responses of three players, drawn randomly from a database of participants involved in a previous run of the same Public Goods game. Each player received 10 euros and could offer some amount of that money (between 0 and 10 euros) to a common pot. The other players offered some amount to the common pot based on the previous data. The content of the common pot was then multiplied by 1.6, and redistributed equally between the players.

## 3. Analytic strategy

All analyses were carried out in R 4.0.0 (https://www.r-project.org/) with R Studio 1.2.5042.

### 3.1. Preliminary analyses

Several preliminary analyses were conducted on the Ipsos dataset immediately after data collection, in 2015 (see Section 5 of the Supplementary Material). Since then, our team has developed more sophisticated analyses to test similar questions and has fully embraced open science practices. The analyses that follow were pre-registered as part of a registered report (osf.io/r5agd).

### 3.2. Multivariate analyses

The dataset was subjected to multivariate analyses through structural equation modelling. The structural equation models were fitted using the R package *lavaan* (Rosseel, 2012). A weighted least-square estimator (WLSMV) was used because it is robust to departures from normality (Rosseel, 2012). Structural equation models involve two major parts: a ‘measurement’ model and a ‘structural’ model.

#### 3.2.1. Specification of the measurement model

Childhood environmental adversity was assessed as a single emergent variable reflecting the sum of *z*-scores obtained from a comprehensive battery of 15 indicators (Mell et al., 2018). Given that we had no particular hypothesis concerning the separate effects of harshness and unpredictability on life-history strategies, we chose to operationalise environmental adversity holistically and aggregate items pertaining to both dimensions into one composite standardised variable. Furthermore, the items were aggregated because adverse childhood events can be seen as risk factors that are not necessarily correlated with one another (Brumbach et al., 2009), but that all contribute to the cumulative probability of developing a particular outcome (in our case an increased investment in reproduction or somatic maintenance). Finally, it is a widespread practice to examine the additive effects of multiple stressors, assuming that the more stressors a child is exposed to, the more their developmental capabilities are negated (Belsky & Fearon, 2002; Sameroff et al., 1987).

Although we combined harsh and unpredictable indicators in the main analysis, we performed an additional analysis where the 15 environmental adversity indicators were divided into two dimensions (i.e. harshness and unpredictability). Items relating to extrinsic mortality, morbidity and socioeconomic status were *z*-scored and summed into a single ‘Childhood harshness’ score. Items relating to parental involvement, parental investment, parental predictability and safety and security were *z*-scored and summed into a single ‘Childhood unpredictability’ score (Glynn et al., 2019; Griskevicius et al., 2011; Pepper & Nettle 2017; Simpson et al., 2012; see Table S4 of the Supplementary Material for the division of items).

Life-history strategy was modelled as a latent variable aiming to capture the covariations of eight indicators (Mell et al., 2018). There is evidence that indicators relating to somatic maintenance are not straightforwardly related to life-history phenotypes. Brown and Sear (2020) found that health behaviours (e.g. general health, smoking, alcohol consumption) do not cluster well with other life-history clusters (i.e. reproduction and parenting). Therefore, we performed an additional analysis without the life-history strategy indicators relating to somatic maintenance (i.e. body mass index (BMI), smoking and health). This new model is represented in Figure S1 of the Supplementary Material.

Cooperation was modelled as a latent variable aiming to capture the covariations of the three economic games. Peysakhovich et al. (2014), indeed found evidence for a so-called ‘cooperative phenotype’ with highly correlated behaviour across various economic games,

#### 3.2.2. Specification of the structural model

The structural part of the model allowed us to test the following direct and indirect associations: (a) childhood adversity and cooperation; (b) childhood adversity and life-history strategy; (c) life-history strategy and cooperation; and (d) childhood adversity and cooperation via life-history strategy. The latent variables life-history strategy and cooperation were scaled by fixing their variance to 1. Finally, the correlations between the residual errors of the reproduction life-history strategy indicators and the residual errors of the somatic maintenance life-history strategy indicators were included separately in the model, which is also suggested by Mell et al. (2018). This was done because we expected that they showed some additional degree of correlation that was not captured by a single general factor. For example, participants suffering from hereditary diseases probably tend to declare a poorer health status and higher efforts in looking after their health, even though this might not be linked to investments in reproduction. The model's implied covariance matrix therefore captures correlations between items that are not explained by the life-history strategy latent variable, but that can still theoretically be expected to correlate owing to various unmeasured causes. The full model is represented in the results section (Figure 2).

#### 3.2.3. Covariates

Given that cooperation is affected by age, we included age as an auxiliary variable to control for its effect on the cooperation variables. Freund and Blanchard-Fields (2014), for example, found that older adults report valuing contributions to the public good more positively and are more likely to behave altruistically than younger adults. Furthermore, age is also used as an auxiliary variable to control for its effect on the life-history strategy indicators, because Mell et al. (2018) indeed found that age correlates with all of them.

In an additional analysis, we controlled for the effect of current environmental harshness on the life-history strategy and cooperation indicators. Griskevicius et al. (2011) found that childhood environmental harshness and current environmental harshness are moderately correlated (*r* = 0.41). Lettinga et al. (2020) found that childhood adversity and current adversity are both uniquely related to cooperation. The relationship between childhood adversity and cooperation even remained significant when the effect of current adversity was controlled for. Following Griskevicius et al. (2011), current environmental harshness was measured using the following three indicators: ‘I have enough money to buy things I want’, ‘I don't need to worry too much about paying my bills’ and ‘I don't think I'll have to worry about money too much in the future’ (scale 0–100), which was averaged into a single index.

#### 3.2.4. Assessing the model's fit

In order to test the model's fit we used the scaled version of the Comparative Fit Index (*CFI.scaled*), the scaled version of the Root Mean Square Error of Approximation (*RMSEA.scaled*) and the Standardised Root Mean Square Residual (*SRMR*), which is recommended by Hooper et al. (2008). A good fit is commonly assumed if the indices are close to the following values: CFI > 0.95, RMSEA < 0.07 and SRMR < 0.08.

### 3.3. Missing data

Although our indicators of interest showed overall low percentages of missing responses (ranging from 0 to 6%), multiple imputation techniques were used to preserve sample size and avoid biased estimations of model parameters. For the imputed data, 20 complete datasets were generated by fully conditional specifications for categorical and continuous data using the R package *mice* (Buuren & Groothuis-Oudshoorn, 2010). Different imputation methods were used depending on the type of missing data. Predictive mean matching was used for numeric indicators, logistic regression imputation for binary data and proportional odds model for ordered categorical indicators with more than two levels. The function *runMI* of the R package *semTools* (SemTools, 2016) was used to combine the results obtained for the 20 imputed datasets.

### 3.4. Testing the capacity of the model to predict unknown data using stratified k-fold cross-validation

One of the main advantages of structural equation modelling is that it is specifically designed to account for complex, multivariate data but these models are also at risk of overfitting. Overcoming this risk is necessary to ensure the validity of a model, although this is too rarely done in the existing structural equation modelling literature (MacCallum et al., 1992). In the present study, we used a *k*-fold cross-validation approach to rule out overfitting and assessed the capacity of our model to predict unknown data. Without cross-validation one can only have information on how the model performs to the in-sample data (i.e. data where the model is based on). Cross-validation allowed us to assess the predictive performance of the model and to see how the model performed in terms of accuracy of its predictions on a new dataset. Beyond measuring a model's predictive accuracy, the other advantage of cross-validation is that it subjects the model to sampling variability and therefore allows estimating its stability across multiple reshuffling and re-stratification of the data. Cross-validation analyses were performed following six steps, each detailed in Section 8 of the Supplementary Material.

### 3.5. Mediation analyses

A recent paper (MacKinnon et al., 2004) recommends the use of the non-parametric bootstrapping method by Preacher and Hayes (2008) to estimate mediation effects. The main feature of this test is that it does not rely on the assumption of normality. However, this method is computationally costly and cannot be easily integrated with the cross-validation approach. Therefore, two methods for mediation analyses were applied. First, the indirect effect was estimated for each cross-validation iteration using a computationally cheaper method (i.e. Delta method). Second, to confirm its reliability, we also applied the recommended procedure by Preacher and Hayes (2008) mentioned above. We computed bootstrapped 95% confidence intervals (1,000 bootstrap samples) for each of the 20 imputed datasets and then took the average of these datasets.

## 4. Positive control checks

To determine if our sample is representative in terms of the distribution of the initial contribution in economic games, our data was compared with what is considered standard in the behavioural economics literature. For the Dictator game, participants give a mean amount between 20 and 30%, and the modal offer is typically 0% (Engel, 2011). For the Trust game, trustors give a mean amount of about 50% (Johnson & Mislin, 2011). For the Public Goods game, participants contribute a mean amount between 40 and 60%, although there is a wide variance, with most contributing either everything or nothing (Zelmer, 2003).

Furthermore, we included additional analyses on a self-reported measure of social trust. We used three questions from the European Values Study and the European Social Survey, which are among the most commonly used questions to measure social trust in the literature (Glaeser et al., 2000; ‘Generally speaking, would you say that most people can be trusted or that you can't be too careful in dealing with people?’, ‘Would you say that people usually only take care of themselves or that they try to be helpful most of the time?’ and ‘Do you think that most people would try to take advantage of you if they had the opportunity or that they would try to be fair?’). Individual scores on the self-reported trust items were *z*-scored and summed into a single ‘Social trust’ score. Our first analysis tested whether there was a correlation between self-reported social trust scores and the mean transfer in the Trust game. In a second analysis, we ran the structural equation model but replaced the economic games with this measure of social trust. The estimated parameters, fit indices and cross-validation indices were used to check whether the model including self-reported social trust differed from the model including the economic games. If we find a null result with the main analysis using economic games but not with self-reported trust, this will work as suggestive evidence that the mixed results in the literature can be partly traced back to differences in instruments.

## 5. Descriptive statistics

### 5.1. Distributions economic games contributions

**Figure 1.**
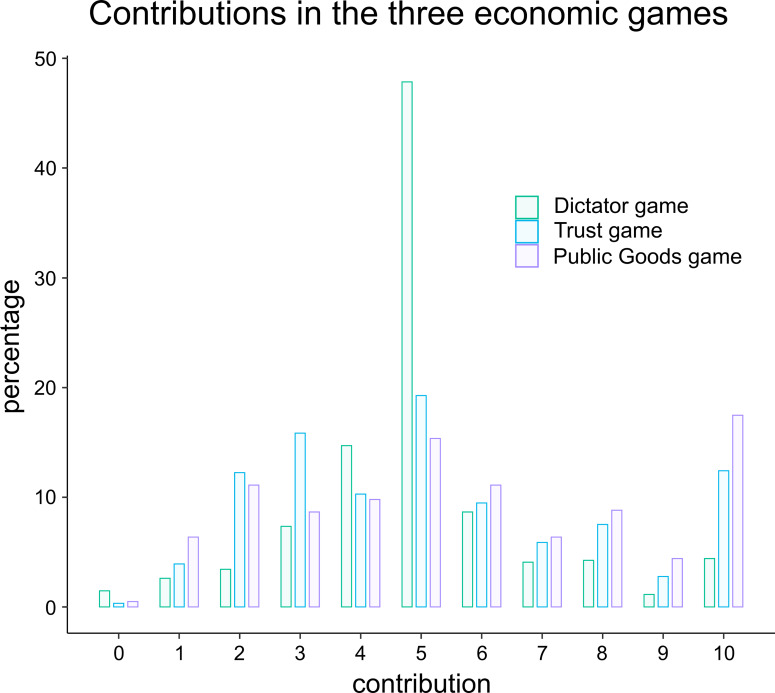
Distribution of contributions (in percentages) per economic game. The initial contributions for the Dictator game are in green, those for the Trust game in blue and those for the Public Goods game in purple.

The distribution of the economic games contributions can be found in Figure 1. In our sample, the mean initial contribution for the Dictator game is 5 euros out of 10, with a high peak at 5 and a distribution that is slightly left-skewed. In experiments, which usually involve students, the average offer usually falls in the 20–30% range (Engel, 2011). However, in non-student populations like the one tested in our study, the average offer is closer to 50% (Engel, 2011). Thus, our distribution is representative of a non-student population.

The mean initial contribution in our Trust game is 5.2 euros out of 10, with a high peak at 5 and a distribution that is slightly left-skewed. In line with this distribution, prior work has shown that trustors typically send about 50% of what they have (Johnson & Mislin, 2011).

The mean initial contribution in our Public Goods game is 5.7 euros out of 10, with high peaks at 10 and 5 and a distribution that is right-skewed. Prior work has shown that students in one-shot Public Goods games contribute on average a mean amount between 40% and 60%, although there is a wide variance, with most contributing either everything or nothing (Zelmer, 2003). In this particular game, our population is on the generous side of the spectrum.

### 5.2. Correlation matrix and descriptive statistics

The correlation matrix and descriptive statistics (i.e. mean, median, standard deviation, range) for the variables included in the main structural equation model and self-reported social trust (based on the whole sample) can be found in Table 1.

**Table 1.** Correlation matrix and descriptive statistics

	(1)	(2)	(3)	(4)	(5)	(6)	(7)	(8)	(9)	(10)	(11)	(12)	(13)	(14)
(1) Childhood adversity	—													
(2) BMI	0.01	—												
(3) Smoking	0.07	0.06	—											
(4) Health effort	−0.08	−0.06	−0.14*	—										
(5) Health status	−0.14*	−0.17*	−0.06	0.16*	—									
(6) Number of children	0.07	0.12*	−0.08	0.06	−0.02	—								
(7) Age at first birth	−0.15*	−0.05	−0.04	−0.09*	0.14*	−0.38*	—							
(8) Sexual debut	−0.08	−0.01	−0.28*	0.07	−0.01	0.03	0.20*	—						
(9) Short-term partners	0.03	0.11*	0.23*	−0.02	−0.02	−0.09*	0.13*	−0.32*	—					
(10) Dictator game	0.02	−0.05	0.07	0.04	−0.06	0.04	−0.01	0.00	0.03	—				
(11) Trust game	−0.02	0.07	0.01	−0.01	−0.06	−0.02	0.06	0.02	0.04	0.30*	—			
(12) Public Goods game	−0.03	0.03	0.02	−0.02	−0.04	−0.02	0.04	−0.02	0.07	0.21*	0.35*	—		
(13) Age	−0.04	0.15*	0.04	0.27*	−0.18*	0.24*	−0.20*	0.27*	0.00	0.05	−0.01	−0.03	—	
(14) Social trust	−0.05	0.03	−0.08	0.15*	0.02	0.07	−0.09*	0.03	−0.03	0.05	0.00	0.05	0.20*	—
Median	0.17	25.46	0.13	71.00	3.00	2.00	25.00	18.00	3.00	5.00	5.00	5.00	56.00	−0.01
Mean	0.21	26.06	0.23	68.95	2.75	2.18	25.45	18.68	6.56	4.99	5.21	5.67	52.75	0.00
SD	0.14	5.13	0.25	18.90	0.69	0.94	4.77	3.44	12.69	1.86	2.66	2.90	14.49	0.99
Range	0–1	12–48	0–1	0–100	1–4	1–6	14–46	13–61	0–160	0–10	0–10	0–10	19–83	−2.59–2.83

* *p* < 0.05.

The raw correlation matrix shows that the childhood environmental adversity score is correlated with two out of the eight life-history strategy indicators and none of the cooperation indicators. Furthermore, it shows low but significant correlations among half of the life-history strategy indicators, the direction of the effects is in most cases consistent with our predictions. Finally, it shows that all three economic games are correlated.

## 6. Multivariate analyses

Parameters (i.e. regression weights, factor loadings), statistics (i.e. *z* statistics, *p*-values) and fit indices (i.e. *CFI.scaled*, *RMSEA.scaled*, *SRMR*) of a given structural equation model are expressed in terms of the median. The reason is that medians are more accurate than means in accounting for the model's precision in the present context; the distributions of values are often skewed because of the sampling variability resulting from the multiple re-shuffling and re-stratification of data during the cross-validation procedure. Cross-validation results are available in Sections 9–13 of the Supplementary Material.

### 6.1. Main model

#### 6.1.1. Model convergence and fit

For the results described next, cross-validation is used (see Section 8 of the Supplementary Material for a detailed description of the methodology). In 8.3% of the 1,000 cross-validation iterations at least one Heywood case was detected – an indication that the fitting algorithm failed to find a valid statistical solution in at least one imputed dataset. The results that we report below are extracted from the 91.7% of iterations that led to a valid solution among each of the 20 imputed datasets. The scaled *CFI* value (0.970), the scaled *RMSEA* value (0.021) and the *SRMR* value (0.032) are consistent with a close-fitting model and reveal no strong misspecification for this model.

#### 6.1.2. Measurement model

**Figure 2.**
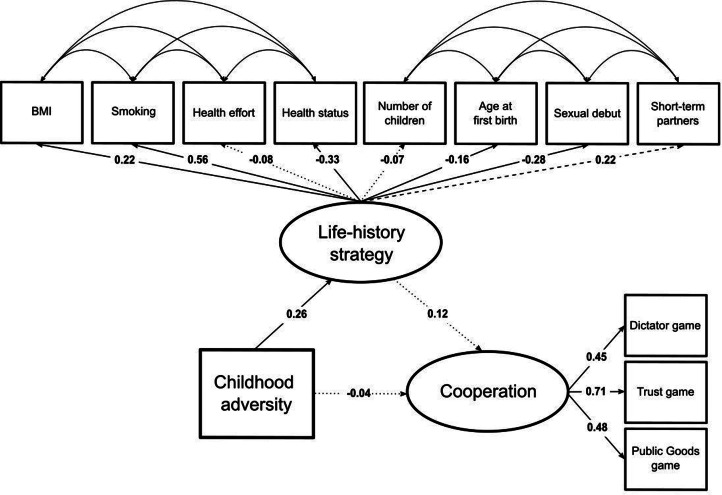
Standardised parameter values estimated by the structural equation model. Significant paths at the 5% level are represented with a continuous arrow, marginally significant paths at the 10% level are represented with a dashed arrow and non-significant paths are represented with a dotted arrow.

The standardised regression weights can be found in Figure 2 (see full results in Section 9 of the Supplementary Material).

‘Dictator game’ (*UnStd.c* = 0.82, *z* = 5.33, *p* = 9.76 × 10^−8^, *Std.c* = 0.45), ‘Trust game’ (*UnStd.c* = 1.88, *z* = 7.78, *p* = 7.26 × 10^−15^, *Std.c* = 0.71) and ‘Public Goods game’ (*UnStd.c* = 1.39, *z* = 6.85, *p* = 7.37 × 10^−12^, *Std.c* = 0.48) loaded significantly on the cooperation latent variable, whose greater values indicate higher initial contributions in the economic games. This confirms hypothesis 1 (i.e. the behavioural measures extracted from the three economic games relate to a single hypothesised latent construct). The average explained variance (*R*^2^) of the individual indicators by the cooperation latent variable is 0.32, which is considered substantial (Cohen, 1988).

Five out of the eight indicators loaded significantly on the life-history strategy latent variable: ‘BMI’ (*UnStd.c* = 0.11, *z* = 2.00, *p* < 0.05, *Std.c* = 0.22), ‘Smoking’ (*UnStd.c* = 0.14, *z* = 3.53, *p* = 4.18 × 10^−4^, *Std.c* = 0.56), ‘Health status’ (*UnStd.c* = −0.22, *z* = −2.56, *p* = 0.01, *Std.c* = −0.33), ‘Age at first child's birth’ (*UnStd.c* = −0.08, *z* = −2.01, *p* = 0.04, *Std.c* = −0.16) and ‘Sexual debut’ (*UnStd.c* = −0.10, *z* = −3.76, *p* = 1.69 × 10^−4^, *Std.c* = −0.28). ‘Short-term partners’ marginally correlated with scores on the life-history strategy latent variable (*UnStd.c* = 0.31, *z* = 1.80, *p* = 0.07, *Std.c* = 0.22). ‘Health effort’ (*p* = 0.49) and ‘Number of children’ (*p* = 0.36) were not captured by the life-history strategy latent variable. The average *R*^2^ of the significant indicators by the life-history strategy latent variable was 0.14, which is considered moderate (Cohen, 1988).

Estimated covariances revealed correlations between some somatic maintenance indicators. Specifically, ‘BMI’ and ‘Health effort’ (*UnStd.c* = −0.12, *z* = −1.98, *p* < 0.05, *Std.c* = −0.13) were negatively associated. ‘Health status’ and ‘Health effort’ (*UnStd.c* = 0.27, *z* = 3.45, *p* = 5.70 × 10^−4^, *Std.c* = 0.23) were positively associated.

Estimated covariances revealed correlations between most of the reproduction indicators. Specifically, ‘Number of children’ and ‘Age at first child's birth’ (*UnStd.c* = −0.14, *z* = −6.36, *p* = 2.08 × 10^−10^, *Std.c* = −0.33) were negatively associated. ‘Age at first child's birth’ and ‘Sexual debut’ (*UnStd.c* = 0.03, *z* = 2.77, *p* < 0.01, *Std.c* = 0.18) were positively associated. ‘Age at first child's birth’ and ‘Short-term partners’ (*UnStd.c* = 0.10, *z* = 2.42, *p* = 0.02, *Std.c* = 0.14) were positively associated. ‘Sexual debut’ and ‘Short-term partners’ (*UnStd.c* = −0.05, *z* = −1.82, *p* = 0.07, *Std.c* = −0.10) were negatively and marginally correlated.

Overall, results of the life-history strategy latent variable are fairly consistent with prior studies (Mell et al., 2018) and partially confirm hypothesis 2 (i.e. individuals’ life-history strategy – the reproduction–maintenance trade-off – can be adequately modelled as a hypothetical latent construct).

#### 6.1.3. Structural model

Figure 2 shows that childhood environmental adversity is not associated (*UnStd.c* = −0.29, *z* = −0.51, *p* = 0.61, *Std.c* = −0.04) with cooperation. Thus, hypothesis 3 (i.e. individuals who experienced greater levels of adversity during their childhood display weaker cooperative tendencies later in life) is not confirmed. Childhood environmental adversity is significantly, albeit moderately, associated with life-history strategy (*UnStd.c* = 1.92, *z* = 3.14, *p* < 0.01, *Std.c* = 0.26). The *R*^2^ of the life-history strategy latent variable by childhood adversity is 0.07, which is considered weak to moderate (Cohen, 1988). Overall, an adverse childhood environment is associated with an increased investment in reproduction and a decreased investment in somatic maintenance. Finally, the association between life-history strategy and cooperation is not significant (*UnStd.c* = 0.12, *z* = 1.30, *p* = 0.19, *Std.c* = 0.12). The *R*^2^ of the cooperation latent variable by childhood adversity and life-history strategy is 0.02, which is considered weak (Cohen, 1988).

The mediation analysis via the Delta method is not significant (indirect effect: *UnStd.c* = 0.23, *z* = 1.05, *p* = 0.29, *Std.c* = 0.03). The mediation analysis via bootstrapping is also not significant (indirect effect: *UnStd.c* = 0.002, *BCI.lower* = −0.000, *BCI.upper* = 0.014, *z* = 0.41, *p* = 0.68). Thus, hypothesis 4 (i.e. the negative association between childhood environmental adversity and cooperation is mediated by individuals’ life-history strategy) is not confirmed.

The pattern remains mostly the same after the inclusion of current environmental harshness as an additional covariate (Heywood cases = 26.1%, scaled *CFI* = 0.974, scaled *RMSEA* = 0.020, *SRMR* = 0.031). The only difference is that ‘BMI’, ‘Health status’ and ‘Age at first child's birth’ do not significantly load on the life-history strategy latent variable anymore and the covariance between ‘BMI’ and ‘Health effort’ turned non-significant (see full results in Section 10 of the Supplementary Material).

### 6.2. Without somatic maintenance

We performed an additional analysis, where we removed the life-history strategy indicators related to somatic maintenance (i.e. BMI, smoking and health). Again, the above-mentioned effects remained largely intact (Heywood cases = 5.1%, scaled *CFI* = 1.000, scaled *RMSEA* = 0.000, *SRMR* = 0.022). The only difference was that all covariance turned non-significant (see full results in Section 11 of the Supplementary Material).

### 6.3. Harshness and unpredictability

We divided childhood environmental adversity into two single composite scores – childhood harshness and childhood unpredictability – in order to test their relative contribution to life-history strategy and cooperation (see Section 3.2.1 of the main text and Table S4 of the Supplementary Material).

#### 6.3.1. Model convergence and fit

As previously described, the results we report are extracted from the 98% of cross-validation iterations that led to a valid solution among each of the 20 imputed datasets. The scaled *CFI* value (0.964), the scaled *RMSEA* value (0.021) and the *SRMR* value (0.033) are consistent with a close-fitting model and reveal no strong misspecification for this model.

#### 6.3.2. Results

When childhood harshness and childhood unpredictability are separated, the overall results are similar to those obtained with the main model (see Section 6.1 of the main text). The single noteworthy difference is that only childhood unpredictability is significantly associated with life-history strategy (*UnStd.c* = 0.21, *z* = 3.26, *p* < 0.01, *Std.c* = 0.26). Overall, greater unpredictability experienced during childhood is linked to greater investments in reproduction and lower investments in somatic maintenance, which is in line with a previous study (Simpson et al., 2012). Childhood harshness, on the other hand, is not significantly associated with life-history strategy (*UnStd.c* = 0.05, *z* = 1.07, *p* = 0.29, *Std.c* = 0.08). This indicates that the significant effect between childhood environmental adversity and life-history strategy found in the main model is mainly driven by the unpredictability indicators (see full results in Section 12 of the Supplementary Material).

**Figure 3.**
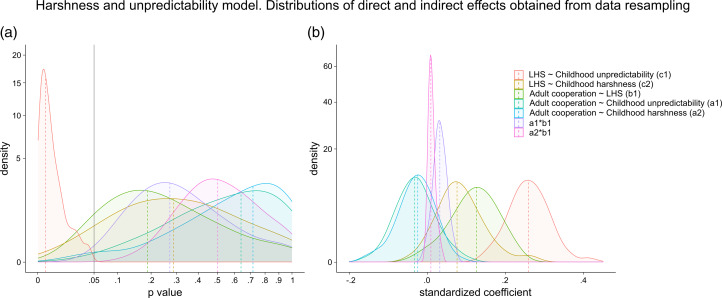
Distribution and medians of the direct and indirect effects for the harshness and unpredictability model. (a) *p*-values. The grey line is the standard alpha level of 0.05. Both axes are squared. (b) Standardised coefficients. The *y*-axis is squared.

Figure 3 displays the distribution and medians of the direct and indirect effects. The only effect that is significant is the relationship between childhood unpredictability and life-history strategy (*p* < 0.01, *Std.c* = 0.26).

### 6.4. Exploratory analyses

In the previous sections we did not find the expected relationship between childhood environmental adversity and cooperation. A possible explanation for this null result is that economic games are a poor predictor of cooperative behaviour (Galizzi & Navarro-Martinez, 2019). In order to test if we can find support for this explanation, we performed several exploratory analyses. The additional analyses are based on the well-known relationship between current environmental adversity and social trust, where more current adversity is associated with decreased levels of social trust (Alesina & La Ferrara, 2002; Brandt et al., 2015; Guillou et al., 2020; Korndörfer et al., 2015; Mell et al., 2020).

#### 6.4.1. Current adversity and self-reported social trust

We first tried to replicate the finding that more current adversity (measured as described in Section 3.2.3) is associated with decreased levels of self-reported social trust (measured as described in Section 4 of the main text). We indeed found that more current adversity is associated with decreased levels of social trust (*UnStd.c* = −0.64, *z* = −3.25, *p* < 0.01, *Std.c* = −0.16), which is consistent with previous findings (Alesina & La Ferrara, 2002; Brandt et al., 2015; Guillou et al., 2020; Korndörfer et al., 2015; Mell et al., 2020). The *R*^2^ of self-reported social trust is 0.06, which is considered weak to moderate (Cohen, 1988).

#### 6.4.2. Current adversity and economic games

Then we tried to replicate the above finding by replacing self-reported social trust with (1) a latent variable based on the three economic games and (2) the Trust game specifically. We found no association between current adversity and cooperation measured using all three economic games (*UnStd.c* = −0.07, *z* = −0.28, *p* = 0.72, *Std.c* = −0.02) or the Trust game considered separately (*UnStd.c* = 0.22, *z* = 0.41, *p* = 0.65, *Std.c* = 0.02). The *R*^2^, of the latent variable based on the three economic games and the Trust game specifically, by current adversity is both null.

These results show that the well-known relationship between current adversity and social trust is present in our sample, but only when social trust is measured via a questionnaire and not when it is measured using economic games. Therefore, the reason for the null result, where childhood environmental adversity is not associated with cooperation, might be due to the use of economic games.

### 6.5. Self-reported social trust

In the registered analyses, we found no direct effect between childhood adversity and cooperation and no indirect effect through life-history strategy. However, since we found a relationship between current environmental adversity and self-reported social trust in the exploratory analyses, we aimed to replicate our main model (see Section 6.1) after having replaced the cooperation latent variable (based on economic games) with self-reported social trust. Therefore, this analysis tested whether childhood environmental adversity negatively correlated with social trust, and whether life-history strategy could mediate such correlation.

#### 6.5.1. Correlation between social trust and the Trust game

Self-reported social trust scores and the initial contributions in the Trust game are not normally distributed (Shapiro–Wilk test: *W* = 0.99, *p* < 0.001 for self-reported social trust; *W* = 0.93, *p* < 0.001 for the Trust game). Therefore, the Spearman rank correlation coefficient is used. The correlation between self-reported social trust scores and Trust game scores is almost null (*ρ* = 0.00, *p* = 0.92).

#### 6.5.2. Model convergence and fit

As previously, the results we report are extracted from the 57% of cross-validation iterations that led to a valid solution among each of the 20 imputed datasets. Compared with the main model and the model where harshness and unpredictability are separated, this model has a lot of problematic iterations even though its number of parameters is equal to the former and inferior to the latter, which is an indication of a low robustness to sampling variability. For the remaining 57% of the valid iterations of the model, the scaled *CFI* value (0.928), the scaled *RMSEA* value (0.040) and the *SRMR* value (0.034) are fairly consistent with a close fit.

#### 6.5.3. Measurement model

**Figure 4.**
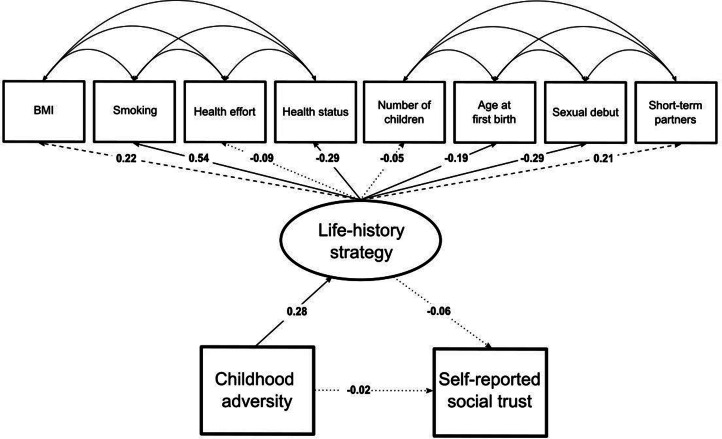
Standardised parameter values estimated by the structural equation model. Significant paths at the 5% level are represented with a continuous arrow, marginally significant paths at the 10% level are represented with a dashed arrow and non-significant paths are represented with a dotted arrow.

The standardised regression weights can be found in Figure 4 (see full results in Section 13 of the Supplementary Material).

Four out of the eight indicators loaded significantly on the life-history strategy latent variable: ‘Smoking’ (*UnStd.c* = 0.13, *z* = 3.58, *p* = 3.42 × 10^−4^, *Std.c* = 0.54), ‘Health status’ (*UnStd.c* = −0.19, *z* = −2.37, *p* = 0.02, *Std.c* = −0.29), ‘Age at first child's birth’ (*UnStd.c* = −0.09, *z* = −2.23, *p* = 0.03, *Std.c* = −0.19) and ‘Sexual debut’ (*UnStd.c* = −0.10, *z* = −3.82, *p* = 1.33 × 10^−4^, *Std.c* = −0.29). ‘BMI’ (*UnStd.c* = 0.11, *z* = 1.92, *p* = 0.06, *Std.c* = 0.22) and ‘Short-term partners’ (*UnStd.c* = 0.30, *z* = 1.72, *p* = 0.09, *Std.c* = 0.21) marginally correlated with scores on the life-history strategy latent variable. ‘Health effort’ (*p* = 0.42) and ‘Number of children’ (*p* = 0.50) were not captured by the life-history strategy latent variable. The average *R*^2^ of the significant indicators by the life-history strategy latent variable is 0.15, which is considered moderate (Cohen, 1988).

Estimated covariances revealed correlations between several somatic maintenance indicators. Specifically, ‘BMI’ and ‘Health effort’ (*UnStd.c* = −0.12, *z* = −1.99, *p* < 0.05, *Std.c* = −0.13) were negatively associated. ‘Health status’ and ‘Health effort’ (*UnStd.c* = 0.27, *z* = 3.39, *p* = 7.07 × 10^−4^, *Std.c* = 0.22) were positively associated. ‘BMI’ and ‘Health status’ (*UnStd.c* = −0.05, *z* = −1.85, *p* = 0.06, *Std.c* = −0.14) were negatively and marginally correlated.

Estimated covariances revealed correlations between most of the reproduction indicators. Specifically, ‘Number of children’ and ‘Age at first child's birth’ (*UnStd.c* = −0.14, *z* = −6.10, *p* = 1.07 × 10^–9^, *Std.c* = −0.33) were negatively associated. ‘Age at first child's birth’ and ‘Sexual debut’ (*UnStd.c* = 0.03, *z* = 2.54, *p* = 0.01, *Std.c* = 0.17) were positively associated. ‘Age at first child's birth’ and ‘Short-term partners’ (*UnStd.c* = 0.10, *z* = 2.37, *p* = 0.02, *Std.c* = 0.15) were positively associated. ‘Sexual debut’ and ‘Short-term partners’ (*UnStd.c* = −0.05, *z* = −1.71, *p* = 0.09, *Std.c* = −0.10) were negatively and marginally correlated.

#### 6.5.4. Structural model

Figure 4 shows that childhood environmental adversity is not associated with self-reported social trust (*UnStd.c* = −0.11, *z* = −0.24, *p* = 0.75, *Std.c* = −0.02). Childhood environmental adversity is once again significantly associated with life-history strategy (*UnStd.c* = 2.08, *z* = 3.25, *p* < 0.01, *Std.c* = 0.28). The *R*^2^ of the life-history strategy latent variable by childhood adversity is 0.08, which is considered weak to moderate (Cohen, 1988). Overall, an adverse childhood environment is associated with an increased investment in reproduction and a decreased investment in somatic maintenance. The association between life-history strategy and self-reported social trust is not significant (*UnStd.c* = −0.05, *z* = −0.72, *p* = 0.47, *Std.c* = −0.06). The *R*^2^ of self-reported social trust by childhood adversity and life-history strategy is 0.05, which is considered weak (Cohen, 1988). Finally, the mediation analysis via the Delta method is not significant either (indirect effect: *UnStd.c* = −0.11, *z* = −0.59, *p* = 0.55, *Std.c* = −0.02).

Thus, even with self-reported social trust (which was the only measure affected by current environmental adversity), the proposed main effect and mediation effect were not found.

## 7. Discussion

The goal of the present study was to test the association between childhood environmental adversity and adult cooperation using economic games in a representative sample of the French population and to test whether the putative effect of adverse childhood environments on cooperation is mediated by differences in individuals’ life-history strategy. Our structural equation models do not verify our main hypotheses: childhood environmental adversity has no direct effect on adult cooperation, or indirect effect through life-history strategy. These results are all the more remarkable because our data are characterised by: (a) a robust pattern of associations between the three economic games and the cooperation latent variable; (b) a robust pattern of associations between the reproduction and somatic maintenance indicators and the life-history strategy latent variable (robust here is used in the sense that the reported parameters are the medians of their distributions obtained under high sampling variability); (c) an association between adverse childhood environments and more short-term reproductive goals as well as fewer long-term health goals; and (d) unpredictability during childhood, rather than harshness, as the driving factor behind the relationship between childhood environmental adversity and life-history-strategy. These associations suggest that the data we collected behaves according to a pattern that corresponds to prior work.

Childhood environmental adversity was not associated with adult cooperation in our models, which is at odds with part of the experimental literature (Lettinga et al., 2020; McCullough et al., 2013; Wu et al., 2020). Two possible, albeit not mutually exclusive, explanations can be put forward: (a) there is no effect between childhood environmental adversity and cooperation; or (b) economic games are a poor predictor of cooperative behaviour. Further analyses showed no association between self-reported social trust and childhood adversity, while a consistent association was found with current environmental adversity, a result which replicates prior findings (Alesina & La Ferrara, 2002; Brandt et al., 2015; Guillou et al., 2020; Korndörfer et al., 2015; Mell et al., 2020). Interestingly, this latter association was not found when social trust was measured using the Trust game or the three economic games combined into a latent variable. To sum up, these results fit both explanations: (a) a true null effect of childhood environmental adversity on cooperation is highly likely in our sample; and (b) economic games are likely to be poor predictors of cooperative behaviours. Although, it is worth noting that the well-known correlation between current environmental adversity and social trust only emerged using the questionnaire data, not using economic games.

Such a discrepancy between economic games and questionnaires is not an isolated finding and raises interesting questions about the validity of economic games to study real-world cooperation (Becker et al., 2012; Boon-Falleur et al., 2020; Chuang & Schechter, 2015; Dang et al., 2020; Lönnqvist et al., 2015). For instance, a recent systematic review and meta-analysis (Galizzi & Navarro-Martinez, 2019) found only weak evidence of a correlation (*r* = 0.14) between economic games and social behaviour in the field. Furthermore, Galizzi and Navarro-Martinez (2019) also performed a large and comprehensive laboratory–field experiment, where the same sample of participants played economic games in the laboratory and were confronted with naturalistic situations related to social preferences in the field, which showed that economic games do a poor job at explaining social behaviours in the field and therefore lack external validity.

A fruitful avenue for future research would be to adjust economic games in order to make them more predictive of real-life cooperation. There are several ways to achieve this goal. First, more contextual features should be added to economic games so that they are more aligned with cooperative behaviour in the field. Much research in psychology and experimental economics has shown that preferences are significantly shaped by the context in which they are elicited (Ariely et al., 2006; Baumard et al., 2013; Gurven & Winking, 2008). For example, Goeschl et al. (2015) found that a Public Goods game with standard parameters was not associated with a specific social behaviour (i.e. voluntary climate change mitigation) measured in another task. However, when the game parameters were more aligned with the social behaviour being studied, they were associated. Also, Lagarde and Blaauw (2014) found that when recipients in a Dictator game were framed in a similar manner (compared with a standard way) to the social behaviour being studied (i.e. taking jobs in rural areas), economic games decisions are linked to real-life behaviour. Thus, by including contextual features in economic games so that they more resemble the social behaviour being studied, generalisability is possible (Camerer, 2011).

Second, there is increasing evidence that economic games decisions are less stable and less predictive of real-life behaviour than responses to questionnaires (Chuang & Schechter, 2015; Frey et al., 2017; Lönnqvist et al., 2015). A possible explanation proposed by Palminteri and Chevallier (2018) is that economic games decisions in the laboratory are probably influenced by the current situation (i.e. state) that the participant is in, while questionnaires are specifically designed to measure people's average behaviour across a long period of time (i.e. state). Therefore, it would be interesting to investigate what happens when people are asked to play several economic games over an extended period of time, so that their average response more closely resembles their true propensity for cooperative behaviour.

Going back to our main results, a number of limitations should be acknowledged and may partially explain why the predicted association was not found. First, the childhood environmental adversity construct is based on retrospective self-reporting by participants in order to synthesise a picture of childhood environmental adversity. Therefore, it is possible that these reports are sensitive to cognitive biases in general and memory biases specifically. There is indeed controversy around the validity of information about childhood experiences gathered from retrospective reports (Widom & Morris, 1997). However, in a similar study, Brown and Sear (2020) suggest that objective measures of environmental adversity are a more robust predictor of life-history strategy than subjective measures. Importantly for our study, most of the indicators (12 out of 15) included in the environmental adversity construct are objective measures about participants’ childhood (e.g. ‘Did your parents get divorced or separated before the age of 18?’), thereby reducing the reliance on subjective measures. Second, there might be other mechanisms, besides the one put forward in our paper, that can explain the relationship between childhood environmental adversity and adult cooperation. For example, the relationship might be mediated by capital restrictions (Nettle, 2015), waiting costs (Mell et al., 2019) or risk management (Amir et al., 2018). Third, the correlational nature of our data must be acknowledged.

To conclude, recent findings indicate that adverse environments are associated with decreased cooperation and that this effect is partly mediated by differences in individuals’ life-history strategy. In this paper, we set out to replicate and extend these findings by measuring actual cooperative behaviour in three economic games – a Dictator game, a Trust game and a Public Goods game – on a diverse sample of 612 people. Although we found that the cooperation and life-history strategy latent variables were adequately captured by the models, the proposed relationship between childhood environmental adversity and adult cooperation and the mediation effect by life-history strategy were not found.

## Supplementary material

For supplementary material accompanying this paper visit https://doi.org/10.1017/ehs.2021.21. click here to view supplementary material

## Research transparency and reproducibility

This manuscript was first submitted and accepted as a registered report, which can be found at osf.io/r5agd. The raw data, study materials, analysis code, laboratory log and deviations from the registered report can be found at osf.io/g4scw.

## References

[ref1] Akee, R., Copeland, W., Costello, E. J., & Simeonova, E. (2018). How does household income affect child personality traits and behaviors? American Economic Review, 108(3), 775–827. 10.1257/aer.20160133 29568124 PMC5860688

[ref2] Alesina, A., & La Ferrara, E. (2002). Who trusts others? Journal of Public Economics, 85(2), 207–234. 10.1016/S0047-2727(01)00084-6

[ref3] Algan, Y., Benkler, Y., Fuster Morell, M., & Hergueux, J. (2013). Cooperation in a peer production economy experimental evidence from Wikipedia. (SSRN Scholarly Paper no. 2843518). Social Science Research Network. https://papers.ssrn.com/abstract=2843518

[ref4] Amir, D., Jordan, M. R., & Rand, D. G. (2018). An uncertainty management perspective on long-run impacts of adversity: The influence of childhood socioeconomic status on risk, time, and social preferences. Journal of Experimental Social Psychology, 79, 217–226. 10.1016/j.jesp.2018.07.014

[ref5] André, J.-B., & Rousset, F. (2019). Does extrinsic mortality accelerate the pace of life? A bare-bones approach. BioRxiv, 777698. 10.1101/777698

[ref6] Andreoni, J., Nikiforakis, N., & Stoop, J. (2017). Are the rich more selfish than the poor, or do they just have more money? A natural field experiment. National Bureau of Economic Research, w23229. 10.3386/w23229

[ref7] Ariely, D., Loewenstein, G., & Prelec, D. (2006). Tom Sawyer and the construction of value. Journal of Economic Behavior & Organization, 60(1), 1–10. 10.1016/j.jebo.2004.10.003

[ref8] Axelrod, R., & Hamilton, W. D. (1981). The evolution of cooperation. Science, 211(4489), 1390–1396. 10.1126/science.7466396 7466396

[ref9] Baumard, N., André, J.-B., & Sperber, D. (2013). A mutualistic approach to morality: The evolution of fairness by partner choice. Behavioral and Brain Sciences, 36(1), 59–78. 10.1017/S0140525X11002202 23445574

[ref10] Becker, A., Deckers, T., Dohmen, T., Falk, A., & Kosse, F. (2012). The relationship between economic preferences and psychological personality measures. Annual Review of Economics, 4(1), 453–478. 10.1146/annurev-economics-080511-110922

[ref11] Belsky, J., & Fearon, R. M. P. (2002). Infant-mother attachment security, contextual risk, and early development. Development and Psychopathology, 14, 293–310. 10.1017/S0954579402002067 12030693

[ref12] Boon-Falleur, M., Bouguen, A., Charpentier, A., Algan, Y., Huillery, E., & Chevallier, C. (2020). Measuring socio-emotional skills in schools: Simple questionnaires outperform behavioral tasks. *Psyarxiv*. 10.31234/osf.io/w7pr6 PMC8748826 35013410

[ref13] Brandt, M. J., Wetherell, G., & Henry, P. J. (2015). Changes in income predict change in social trust: A longitudinal analysis. Political Psychology, 36(6), 761–768. 10.1111/pops.12228

[ref14] Brown, L. J., & Sear, R. (2020). Do parenting, reproductive and health traits cluster together in distinct trajectories? Evidence from two UK cohort studies. 10.31219/osf.io/r8jvw

[ref15] Brumbach, B. H., Figueredo, A. J., & Ellis, B. J. (2009). Effects of harsh and unpredictable environments in adolescence on development of life history strategies. Human Nature, 20(1), 25–51. 10.1007/s12110-009-9059-3 20634914 PMC2903759

[ref16] Bulley, A., & Pepper, G. V. (2017). Cross-country relationships between life expectancy, intertemporal choice and age at first birth. Evolution and Human Behavior, 38(5), 652–658. 10.1016/j.evolhumbehav.2017.05.002

[ref17] Buuren, S. van, & Groothuis-Oudshoorn, K. (2010). mice: Multivariate imputation by chained equations in R. Journal of Statistical Software, 45(3), 1–67. 10.18637/jss.v045.i03

[ref18] Camerer, C. F. (2011). Behavioral game theory: Experiments in strategic interaction. Princeton University Press.

[ref19] Chuang, Y., & Schechter, L. (2015). Stability of experimental and survey measures of risk, time, and social preferences: A review and some new results. Journal of Development Economics, 117, 151–170. 10.1016/j.jdeveco.2015.07.008 30078930 PMC6070154

[ref20] Cohen, J. (1988). Statistical Power Analysis for the Behavioral Sciences. Laurence Erlbaum Associates.

[ref21] Copping, L. T., Campbell, A., & Muncer, S. (2014). Psychometrics and life history strategy: The structure and validity of the high K strategy scale. Evolutionary Psychology, 12(1), 147470491401200130. 10.1177/14747049140120011525299760

[ref22] Dang, J., King, K. M., & Inzlicht, M. (2020). Why are self-report and behavioral measures weakly correlated? Trends in Cognitive Sciences, 24(4), 267–269. 10.1016/j.tics.2020.01.007 32160564 PMC7977810

[ref23] Del Giudice, M., Gangestad, S. W., & Kaplan, H. S. (2015). Life history theory and evolutionary psychology. In D. M. Buss (Ed.), The handbook of evolutionary psychology. Vol. 1. Foundations (pp. 88–114). John Wiley & Sons.

[ref24] Ellis, B. J., & Del Giudice, M. (2019). Developmental adaptation to stress: An evolutionary perspective. Annual Review of Psychology, 70, 111–139. 10.1146/annurev-psych-122216-011732 30125133

[ref25] Ellis, B. J., Figueredo, A. J., Brumbach, B. H., & Schlomer, G. L. (2009). Fundamental dimensions of environmental risk. Human Nature, 20(2), 204–268. 10.1007/s12110-009-9063-7 25526958

[ref26] Engel, C. (2011). Dictator games: A meta study. Experimental Economics, 14(4), 583–610. 10.1007/s10683-011-9283-7

[ref27] Freund, A. M., & Blanchard-Fields, F. (2014). Age-related differences in altruism across adulthood: Making personal financial gain versus contributing to the public good. Developmental Psychology, 50(4), 1125–1136. 10.1037/a0034491 24059256

[ref28] Frey, R., Pedroni, A., Mata, R., Rieskamp, J., & Hertwig, R. (2017). Risk preference sharesthe psychometric structure of major psychological traits. Science advances, 3(10), e1701381. 10.1126/sciadv.1701381 PMC5627985 28983511

[ref29] Galizzi, M. M., & Navarro-Martínez, D. (2019). On the external validity of social preference games: a systematic lab-field study. Management Science, 65(3), 976–1002. 10.1287/mnsc.2017.2908

[ref30] Glaeser, E. L., Laibson, D. I., Scheinkman, J. A., & Soutter, C. L. (2000). Measuring trust. The Quarterly Journal of Economics, 115(3), 811–846. 10.1162/003355300554926

[ref31] Glynn, L. M., Stern, H. S., Howland, M. A., Risbrough, V. B., Baker, D. G., Nievergelt, C. M., … & Davis, E. P. (2019). Measuring novel antecedents of mental illness: The questionnaire of unpredictability in childhood. Neuropsychopharmacology, 44(5), 876–882. 10.1038/s41386-018-0280-9 30470840 PMC6461958

[ref32] Goeschl, T., Kettner, S. E., Lohse, J., & Schwieren, C. (2015). What do we learn from public good games about voluntary climate action? Evidence from an artefactual field experiment. 10.2139/ssrn.2620229

[ref33] Griskevicius, V., Tybur, J. M., Delton, A. W., & Robertson, T. E. (2011). The influence of mortality and socioeconomic status on risk and delayed rewards: A life history theory approach. Journal of Personality and Social Psychology, 100(6), 1015–1026. 10.1037/a0022403 21299312 PMC3298774

[ref34] Guillou, L., Grandin, A., & Chevallier, C. (2020). Correcting misperceptions of relative income: Impact on temporal discounting and social trust. *PsyArXiv*. 10.31234/osf.io/vwyfn

[ref35] Gurven, M., & Winking, J. (2008). Collective action in action: Prosocial behavior in and out of the laboratory. American Anthropologist, 110(2), 179–190. 10.1111/j.1548-1433.2008.00024.x

[ref36] Holland, J., Silva, A. S., & Mace, R. (2012). Lost letter measure of variation in altruistic behaviour in 20 neighbourhoods. PLOS ONE, 7(8), e43294. 10.1371/journal.pone.0043294 PMC3419711 22905250

[ref37] Hooper, D., Coughlan, J., & Mullen, M. (2008). Structural equation modelling: Guidelines for determining model fit. Electronic Journal of Business Research Methods, 6(1), 53–60. 10.21427/D7CF7R

[ref38] INSEE (2018). Life expectancy by standard of living: In men, 13 years of difference between the most affluent and the most modest. No. 1687. https://www.insee.fr/en/statistiques/3533552/

[ref39] Jackson, D. L. (2003). Revisiting sample size and number of parameter estimates: Some support for the N:q hypothesis. Structural Equation Modeling, 10(1), 128–141. 10.1207/S15328007SEM1001_6

[ref40] Jasienska, G., Bribiescas, R. G., Furberg, A.-S., Helle, S., & Núñez-de la Mora, A. (2017). Human reproduction and health: An evolutionary perspective. The Lancet, 390(10093), 510–520. 10.1016/S0140-6736(17)30573-1 28792413

[ref41] Johnson, N. D., & Mislin, A. A. (2011). Trust games: A meta-analysis. Journal of Economic Psychology, 32(5), 865–889. 10.1016/j.joep.2011.05.007

[ref42] Kline, R. B. (2015). Principles and practice of structural equation modeling (4th ed.). Guilford Publications.

[ref43] Korndörfer, M., Egloff, B., & Schmukle, S. C. (2015). A large scale test of the effect of social class on prosocial behavior. PLOS ONE, 10(7), e0133193. 10.1371/journal.pone.0133193 PMC4507988 26193099

[ref44] Lagarde, M., & Blaauw, D. (2014). Pro-social preferences and self-selection into jobs: Evidence from South African nurses. Journal of Economic Behavior & Organization, 107, 136-152. 10.1016/j.jebo.2014.09.004

[ref45] Lettinga, N., Jacquet, P. O., André, J.-B., Baumard, N., & Chevallier, C. (2020). Environmental adversity is associated with lower investment in collective actions. PloS One, 15(7), e0236715. 10.1371/journal.pone.0236715 PMC7392252 32730312

[ref46] Levitt, S. D., & List, J. A. (2007). What do laboratory experiments measuring social preferences reveal about the real world?. Journal of Economic perspectives, 21(2), 153-174. 10.1257/jep.21.2.153

[ref47] Lönnqvist, J.-E., Verkasalo, M., Walkowitz, G., & Wichardt, P. C. (2015). Measuring individual risk attitudes in the lab: Task or ask? An empirical comparison. Journal of Economic Behavior & Organization, 119, 254–266. 10.1016/j.jebo.2015.08.003

[ref48] MacCallum, R. C., Roznowski, M., & Necowitz, L. B. (1992). Model modifications in covariance structure analysis: The problem of capitalization on chance. Psychological Bulletin, 111(3), 490–504. 10.1037/0033-2909.111.3.490 16250105

[ref49] MacKinnon, D. P., Lockwood, C. M., & Williams, J. (2004). Confidence limits for the indirect effect: Distribution of the product and resampling methods. Multivariate Behavioral Research, 39(1), 99–128. 10.1207/s15327906mbr3901_4 20157642 PMC2821115

[ref50] McAuliffe, W. H., Forster, D. E., Pedersen, E. J., & McCullough, M. E. (2019). Does cooperation in the laboratory reflect the operation of a broad trait?. European Journal of Personality, 33(1), 89-103. 10.1002/per.2180

[ref51] McCullough, M. E., Pedersen, E. J., Schroder, J. M., Tabak, B. A., & Carver, C. S. (2013). Harsh childhood environmental characteristics predict exploitation and retaliation in humans. Proceedings of the Royal Society B: Biological Sciences, 280(1750), 20122104. 10.1098/rspb.2012.2104 PMC3574429 23118435

[ref52] Mell, H., Baumard, N., & André, J.-B. (2019). Time is money. Waiting costs explain why selection favors steeper time discounting in deprived environments. *Ecoevorxiv*. 10.32942/osf.io/7d56s

[ref53] Mell, H., Safra, L., Algan, Y., Baumard, N., & Chevallier, C. (2018). Childhood environmental harshness predicts coordinated health and reproductive strategies: A cross-sectional study of a nationally representative sample from France. Evolution and Human Behavior, 39(1), 1–8. 10.1016/j.evolhumbehav.2017.08.006

[ref54] Mell, H., Safra, L., Demange, P., Algan, Y., Baumard, N., & Chevallier, C. (2020). Early life adversity is associated with diminished social trust in adults. *Psyarxiv*. 10.31234/osf.io/43q8z

[ref55] Nettle, D. (2010). Dying young and living fast: Variation in life history across English neighborhoods. Behavioral Ecology, 21(2), 387–395. 10.1093/beheco/arp202

[ref56] Nettle, D. (2011). Flexibility in reproductive timing in human females: Integrating ultimate and proximate explanations. Philosophical Transactions of the Royal Society B: Biological Sciences, 366(1563), 357–365. 10.1098/rstb.2010.0073 PMC3013465 21199840

[ref57] Nettle, D. (2015). Tyneside neighbourhoods: Deprivation, social life and social behaviour in one British city. Open Book Publishers. 10.11647/OBP.0084

[ref58] Nettle, D., Coall, D. A., & Dickins, T. E. (2010). Birthweight and paternal involvement predict early reproduction in British women: Evidence from the National Child Development Study. *American Journal of Human Biology:* The Official Journal of the Human Biology Association, 22(2), 172–179. 10.1002/ajhb.20970 19670389

[ref59] Nettle, D., & Cockerill, M. (2010). Development of social variation in reproductive schedules: A study from an English urban area. PLoS One, 5, e12690. 10.1371/journal.pone.0012690. 20856795 PMC2939869

[ref60] Nettle, D., Colléony, A., & Cockerill, M. (2011). Variation in cooperative behaviour within a single city. PLOS ONE, 6(10), e26922. 10.1371/journal.pone.0026922 PMC3203179 22046411

[ref61] Nowak, M., & Highfield, R. (2011). SuperCooperators: Altruism, evolution, and why we need each other to succeed. Simon and Schuster.

[ref62] Olderbak, S., Gladden, P., Wolf, P. S. A., & Figueredo, A. J. (2014). Comparison of life history strategy measures. Personality and Individual Differences, 58, 82–88. 10.1016/j.paid.2013.10.012

[ref63] Palminteri, S., & Chevallier, C. (2018). Can we infer inter-individual differences in risk-taking from behavioral tasks? Frontiers in Psychology, 9(2307). 10.3389/fpsyg.2018.02307 PMC6260002 30519209

[ref64] Pepper, G. V., & Nettle, D. (2014). Perceived extrinsic mortality risk and reported effort in looking after health. Human Nature, 25(3), 378–392. 10.1007/s12110-014-9204-5 24990431

[ref65] Pepper, G. V., & Nettle, D. (2017). The behavioural constellation of deprivation: Causes and consequences. Behavioral and Brain Sciences, 40, 1–72. 10.1017/S0140525X1600234X 28073390

[ref66] Peysakhovich, A., Nowak, M. A., & Rand, D. G. (2014). Humans display a ‘cooperative phenotype’ that is domain general and temporally stable. Nature Communications, 5, 4939. 10.1038/ncomms5939 25225950

[ref67] Piff, P. K., Kraus, M. W., Côté, S., Cheng, B. H., & Keltner, D. (2010). Having less, giving more: The influence of social class on prosocial behavior. Journal of Personality and Social Psychology, 99(5), 771. 10.1037/a0020092 20649364

[ref68] Preacher, K. J., & Coffman, D. L. (2006). Computing power and minimum sample size for RMSEA. [Computer software.] http://quantpsy.org.

[ref69] Preacher, K. J., & Hayes, A. F. (2008). Asymptotic and resampling strategies for assessing and comparing indirect effects in multiple mediator models. Behavior Research Methods, 40(3), 879–891. 10.3758/BRM.40.3.879 18697684

[ref70] Promislow, D. E. L., & Harvey, P. H. (1990). Living fast and dying young: A comparative analysis of life-history variation among mammals. Journal of Zoology, 220(3), 417–437. 10.1111/j.1469-7998.1990.tb04316.x

[ref71] Rand, D. G., & Nowak, M. A. (2013). Human cooperation. Trends in Cognitive Sciences, 17(8), 413–425. 10.1016/j.tics.2013.06.003 23856025

[ref72] Reznick, D., & Endler, J. A. (1982). The impact of predation on life history evolution in Trinidadian Guppies (*Poecilia reticulata*). Evolution, 36(1), 160–177. 10.1111/j.1558-5646.1982.tb05021.x 28581096

[ref73] Rosseel, Y. (2012). Lavaan: An R package for structural equation modeling. Journal of Statistical Software, 48(2), 1–36. 10.18637/jss.v048.i02

[ref74] Safra, L., Tecu, T., Lambert, S., Sheskin, M., Baumard, N., & Chevallier, C. (2016). Neighborhood deprivation negatively impacts children's prosocial behavior. Frontiers in Psychology, 7. 10.3389/fpsyg.2016.01760 PMC5107739 27895603

[ref75] Sameroff, A. J., Seifer, R., Barocas, R., Zax, M., & Greenspan, S. (1987). Intelligence quotient scores of 4-year-old children: Social–environmental risk factors. Pediatrics, 79, 343–350. 3822634

[ref76] Schmukle, S. C., Korndörfer, M., & Egloff, B. (2019). No evidence that economic inequality moderates the effect of income on generosity. Proceedings of the National Academy of Sciences, 116(20), 9790–9795. 10.1073/pnas.1807942116 PMC6525487 31036660

[ref77] SemTools, S. C. (2016). SemTools: Useful tools for structural equation modelling. R Package Version 0.4-14.

[ref78] Silva, A. S., & Mace, R. (2014). Cooperation and conflict: Field experiments in Northern Ireland. Proceedings of the Royal Society B: Biological Sciences, 281(1792), 20141435. 10.1098/rspb.2014.1435 PMC4150329 25143042

[ref79] Silva, A. S., & Mace, R. (2015). Inter-group conflict and cooperation: Field experiments before, during and after sectarian riots in Northern Ireland. Frontiers in Psychology, 6. 10.3389/fpsyg.2015.01790 PMC4661283 26640449

[ref80] Simpson, J. A., Griskevicius, V., Kuo, S. I.-C., Sung, S., & Collins, W. (2012). Evolution, stress, and sensitive periods: The influence of unpredictability in early versus late childhood on sex and risky behavior. Developmental Psychology, 48(3), 674–686. 10.1037/a0027293 22329381

[ref81] Sjåstad, H. (2019). Short-sighted greed? Focusing on the future promotes reputation-based generosity. Judgment and Decision Making, 14(2), 199–213. 10.31234/osf.io/fw3gp

[ref82] Stamos, A., Lange, F., Huang, S. C., & Dewitte, S. (2020). Having less, giving more? Two preregistered replications of the relationship between social class and prosocial behavior. Journal of Research in Personality, 84, 103902. 10.1016/j.jrp.2019.103902

[ref83] Stearns, S. C. (1992). The evolution of life histories. Oxford University Press.

[ref84] Trivers, R. L. (1971). The evolution of reciprocal altruism. The Quarterly Review of Biology, 46(1), 35–57. 10.1086/406755

[ref85] Widom, C. S., & Morris, S. (1997). Accuracy of adult recollections of childhood victimization, part 2: Childhood sexual abuse. Psychological Assessment, 9(1), 34–46. 10.1037/1040-3590.9.1.34

[ref86] Wu, J., Balliet, D., Tybur, J. M., Arai, S., Van Lange, P. A. M., & Yamagishi, T. (2017). Life history strategy and human cooperation in economic games. Evolution and Human Behavior, 38(4), 496–505. 10.1016/j.evolhumbehav.2017.03.002

[ref87] Wu, J., Guo, Z., Gao, X., & Kou, Y. (2020). The relations between early-life stress and risk, time, and prosocial preferences in adulthood: A meta-analytic review. *Evolution and Human Behavior*. 10.1016/j.evolhumbehav.2020.09.001

[ref88] Zelmer, J. (2003). Linear public goods experiments: A meta-analysis. Experimental Economics, 6(3), 299–310. 10.1023/A:1026277420119

