# Supplementary material for: Childhood environmental adversity is not linked to lower levels of cooperative behaviour in economic games
Source: Evol Hum Sci. 2021 Mar 17;3:e29. doi: 10.1017/ehs.2021.21 (PMC10427279; doi:10.1017/ehs.2021.21)
Supplement: Supplementary file 1 [file S2513843X21000219sup001.docx]

**Childhood environmental adversity is not linked to lower levels of cooperative behaviour in economic games**

**Supplementary Materials**

Lettinga, N.^1,@^, Mell, H.^1^, Algan, Y.^3^, *Jacquet, P.O.^1,2^, *Chevallier, C.^1,@^

^1^Laboratoire de Neurosciences Cognitives et Computationnelles (LNC^2^), Département d'Études Cognitives, INSERM U960, École Normale Supérieure, PSL Research University, F-75005 Paris, France

^2^Institut Jean-Nicod, Département d'Études Cognitives, INSERM U8129, École Normale Supérieure, PSL Research University, F-75005 Paris, France

^3^Sciences Po, OFCE, 27 Rue Saint-Guillaume, 75007 Paris, France

* Equal contribution

@ Corresponding authors:

Address: Laboratoire de Neurosciences Cognitives et Computationnelles (LNC^2^), Département d'Études Cognitives, INSERM U960, École Normale Supérieure, PSL Research University, F-75005 Paris, France

Telephone number: +31 614393500

Email: [niels.lettinga@ens.fr](mailto:niels.lettinga@ens.fr), [coralie.chevallier@ens.fr](mailto:coralie.chevallier@ens.fr)

**Contents**

1. Exclusion criteria subjects Page 3

2. Full questionnaire Page 4

3. Questions childhood environmental adversity Page 9

4. Questions life-history strategy Page 10

5. Preliminary analyses Page 11

6. Harshness and unpredictability questions separated Page 12

7. Structural equation model without somatic maintenance Page 14

8. Cross-validation analyses Page 15

9. Results main model Page 17

10. Results controlled for current environmental harshness Page 19

11. Results without somatic maintenance Page 21

12. Results harshness and unpredictability Page 23

13. Results self-reported social trust Page 28

References

**1. Exclusion criteria subjects**

| **Exclusion criteria subjects** |
| --- |
| Mother's death before participant's birth |
| Mother's death after start of study |
| Participant's birth before mother's age 10 |
| Father's death before participant's birth |
| Father's death after start of study |
| Participant's birth before father's age 10 |
| Position as siblings is larger than the number of children of the mother |
| Position as siblings is larger than the number of children of the father |
| Divorce before child's birth |
| Living with stepfather before participant's birth |
| Mentioned physical, psychological or sexual abuse but also mentioned no abuse to another question |
| Did not mention physical, psychological or sexual abuse but also mentioned abuse to another question |
| Mentioned abuse from inside the family but also mentioned no abuse to another question |
| Mentioned abuse from outside the family but also mentioned no abuse to another question |
| Mentioned troubled relatives but also mentioned no troubled relatives to another question |
| Did not mention troubles relatives but also mentioned troubled relatives to another question |
| Claimed to have moved and changed schools more than 50 times before the age of 7 |
| Mentioned death of siblings but does not report any brother or sister |
| Claims to be smaller than 120 cm |
| Claims to weigh less than 25 kg |
| Years smoking longer than participant's age |
| Mentioned having children but indicates 0 as to how many |
| Age at first child's birth is younger than age 14 of the participant |
| Claims to have had sexual intercourse at an age that is older than the participants' age |
| Claims to have had sexual intercourse before the age of 10 |
| Claims to have had more than 300 short-term sexual partners |

**Supplementary Table S1:** Exclusion criteria subjects. Respondents were removed if they gave too many absurd answers (> 3*sd* from the sample mean)

**2. Full questionnaire**

You will now fill out the last questionnaire in session 1. Some of the questions are personal. You are asked these questions so that participants’ responses can be analyzed according to their individual profile and history. We wish to remind you that all of the responses we collect will be analysed completely anonymously. If you would prefer to not respond to some of the questions, you will simply have to tick the box « I don’t want to answer ».

Your childhood (1/2)

1. Sex :

Female / Male

2. Birth year of your mother?

Please select one of the following answers: (*Answers from 1908 to 1990)*

3. Is your mother still alive?

Yes / No

4. Year of death

*Answer this question only if you answered 'No' to « Is your mother still alive? »*

Please write your answer here:

5. Birth year of your father?

Please select one of the following answers: (*Answers from 1908 to 1990)*

6. Is your father still alive?

Yes / No

7.Year of death

*Answer this question only if you answered 'No' to « Is your father still alive? »*

Please write your answer here:

8. How many children did your mother have?

Please select one of the following answers:

I do not know 1 2 3 4 5 6 7 8 9 10 and more

9. Among your mother’s children you are the:

Please select one of the following answers:

I do not know
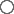
1^st^
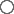
2^nd^
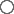
3^rd^
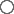
4^th^
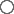
5^th^
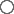
6^th^
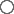
7^th^
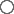
8^th^
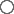
9^th^
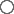
10^th^ or more

10. How many children did your father have?

Please select one of the following answers:

I do not know 1 2 3 4 5 6 7 8 9 10 and more

11. Among your father’s children you are the:

Please select one of the following answers:

I do not know
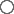
1st
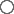
2^nd^
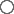
3^rd^
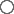
4^th^
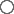
5^th^
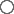
6^th^
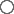
7^th^
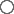
8^th^
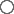
9^th^
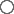
10^th^ or more

12. Did your parents get divorced or separated?

Yes / No

12b. How old were you?

*Answer this question only if you answered 'Yes' to « Did your parents get divorced or separated? »*

Please write your answer here:

13. Have you ever lived with a step-father?

*Answer this question only if you answered 'Yes' to « Did your parents get divorced or separated? »*

Please select one of the following answers:

Yes / No

13b. From what age on?

*Answer this question only if you answered 'Yes' to « Have you ever lived with a step-father? »*

Please write your answer here:

14. Were you ever placed in an institution or in a foster family?

Yes / No

15. My parents always seemed to care about what I was doing.

*Scale from 1 to 100*

16. My father was always there when I needed him.

*Scale from 1 to 100*

17. My mother was always there when I needed her.

*Scale from 1 to 100*

Your childhood (2/2)

18. When I was growing up, someone in my house was always yelling at someone else.

*Scale from 1 to 100*

19. Some of the punishments I received when I was a child now seem too harsh to me.

*Scale from 1 to 100*

20. I guess you could say that I wasn’t treated as well as I should have been at home.

*Scale from 1 to 100*

21. I consider that during my childhood, I was a victim of:

Please select all appropriate answers:

Physical abuse

Sexual abuse

Psychological abuse

No form of abuse

I don’t want to answer

21b. These episodes were caused by:

*Answer this question only if you answered 'Physical abuse' or 'Psychological abuse ' or 'Sexual abuse' to the question «* I consider that during my childhood, I was a victim of *»*

Please select all appropriate answers:

One or several people in my family

One or several people outside my family

22. My family usually had enough money for things when I was growing up.

*Scale from 1 to 100*

23. I grew up in a relatively wealthy neighborhood.

*Scale from 1 to 100*

24. I felt relatively wealthy compared to the other kids in my school.

*Scale from 1 to 100*

25. During your childhood, did you live with one or several people who were:

Please select all appropriate answers:

alcoholic

violent

depressed

who suffered from a mental disorder

who regularly took street drugs

who sometimes had issues with the judicial system

who had spent time in prison

none of the above

I don’t want to answer

26. From your birth until you were 7 years old, did you suffer from a long disease that required a hospitalisation?

Please select one of the following answers:

Yes / No

27. From your birth until you were 7 years old, how many times did you move?

Please write your answer here:

28. From your birth until you were 7 years old, how many times did you change schools?

Please write your answer here:

29. From your birth until you were 7 years old, how much did your father take care of you?

Please select one of the following answers:

He left my mother taking care of us.

He took care of us, but less than my mother.

He took care of us as much as my mother.

He took more care of us than my mother.

This question does not apply to me.

30. From your birth until you were 7 years old, did one of your sibling die?

Yes / No

Your adult life and family

31. How tall are you (in cm)?

Please write your answer here: *(number between 65 and 220)*

32. How heavy are you (in kg)?

Please write your answer here:

33. Have you had children?

Yes / No

33b-1. How many:

*Answer this question only if you answered 'Yes' to « Have you had children? »*

Please write your answer here: *(number between 1 and 19)*

Date of Birth for each child: (*number between 1920 and 2014)*

Your health and safety

34. How much effort do you make to look after your health and ensure your safety these days?

*Scale from 1 to 100*

35. How is your health in general?

Please select one of the following answers:

Bad

Acceptable

Good

Excellent

36. In total, during how many years did you smoke daily or almost daily?

Please write your answer here:

We are now going to ask you some questions about your voluntary sexual experiences (these questions do not apply to non consensual experiences you may have had):

37. Have you ever had a sexual intercourse?

Please select one of the following answers:

Yes / No / I don’t want to answer

38. How old were you when you had your first sexual intercourse?

*Answer this question only if you answered 'Yes' to « Have you ever had sexual intercourse? »*

Please write your answer here:

39. With how many different partners have you had sexual intercourse without having an interest in a long-term committed relationship with this person?

*Answer this question only if you answered 'Yes' to « Have you ever had sexual intercourse? »*

Please write your answer here:

**3. Questions childhood environmental adversity**

|  | **Questions** | **Answers** |
| --- | --- | --- |
|  | General features of the family unit during respondent's childhood |  |
| 1 | Did your parents get divorced or separated before the age of 18? | Yes/ no |
| 2 | Have you ever lived with a stepfather before the age of 18? | Yes/ no |
| 3 | Were you ever placed in an institution or in a foster family? | Yes/ no |
| 4 | Did your mother die before the age of 18? | Yes/ no |
| 5 | Did your father die before the age of 18? | Yes/ no |
| 6 | How many brothers and sisters do you have? |  |
| 7 | From your birth until you were 7 years old, did one of your sibling die? | Yes/ no |
| 8 | "Parental investment" scale |  |
|  | My parents always seemed to care about what I was doing. | 0-100 |
|  | My father was always there when I needed him. | 0-100 |
|  | My mother was always there when I needed her. | 0-100 |
| 9 | "Parenting style" scale |  |
|  | When I was growing up, someone in my house was always yelling at someone else. | 0-100 |
|  | Some of the punishments I received when I was a child now seem too harsh to me. | 0-100 |
|  | I guess you could say that I wasn’t treated as well as I should have been at home. | 0-100 |
|  | Victim of psychological, sexual of psychological abuse during childhood |  |
| 10 | By one or several people in my family | Yes/ no |
| 11 | By one or several people outside my family | Yes/ no |
| 12 | "Family difficulties" index |  |
|  | Alcoholic | Yes/ no |
|  | Violent | Yes/ no |
|  | Depressed | Yes/ no |
|  | Mental disorder | Yes/ no |
|  | Drugs | Yes/ no |
|  | Judicial system | Yes/ no |
|  | Prison | Yes/ no |
| 13 | From your birth until you were 7 years old, did you suffer from a long disease that required hospitalization? | Yes/ no |
| 14 | "Neighbourhood stability" scale |  |
|  | From your birth until you were 7 years old, how many times did you move? |  |
|  | From your birth until you were 7 years old, how many times did you change schools? |  |
| 15 | "Childhood SES" scale |  |
|  | My family usually had enough money for things when I was growing up. | 0-100 |
|  | I grew up in a relatively wealthy neighborhood. | 0-100 |
|  | I felt relatively wealthy compared to the other kids in my school. | 0-100 |

**Supplementary Table S2:** Questions childhood environmental adversity

**4. Questions life-history strategy**

|  | **Questions** |  | **Answers** |
| --- | --- | --- | --- |
|  | Reproduction |  |  |
| 1 | How many children have you had? |  |  |
| 2 | Age at first birth |  | Birth year of the respondent - reported birth year of their first child |
| 3 | How old were you when you had your first sexual intercourse? |  |  |
| 4 | With how many different partners have you had sexual intercourse without having an interest in a long-term committed relationship with this person? |  |  |
|  | Maintenance |  |  |
| 5 | Body mass index (BMI) |  | 10000*how heavy are you (in kg)/ how tall are you (in cm)2 |
| 6 | Level of cigarette’s consumption |  | During how many years did you smoke daily or almost daily/ respondent's age |
| 7 | How is your health in general? |  | Bad/ acceptable/ good/ excellent |
| 8 | How much effort do you make to look after your health and ensure your safety these days? |  | 0-100 |

**Supplementary Table S3:** Questions life-history strategy. In order for the models to converge the variables “BMI”, “Health effort”, “Age at first child’s birth”, “Sexual debut” and “Short-term partners” had to be divided by 10 in order to homogenize the variances between the indicators.

**5. Preliminary analyses**

Using simple regression models, these initial analyses found no significant effect of respondents’ childhood environmental adversity on initial contribution in the Dictator game (*b* = 0.06, *p* = 0.21, *N* = 398), the Trust game (*b* = 0.04, *p* = 0.41, *N* = 398) and the Public Goods game (*b* = 0.02, *p* = 0.56, *N* = 398; *t*(30) = -1.18, *p* = 0.25, *N* = 154). No other associations were tested at that time.

The new analyses differed from the preliminary analyses in several ways. First, we looked at the entire sample. Second, a more comprehensive method was used to calculate childhood environmental adversity. Childhood environmental adversity was measured using the same items and methodology as in Mell et al. (2018). Third, life-history strategy was included as a mediator between childhood environmental adversity and cooperation using the same logic as the one presented in Lettinga et al. (2020). We exploited indicators of life-history strategies that come as close as possible to testing the reproduction-maintenance trade-off (Mell et al., 2018). Fourth, we used structural equation modeling instead of simple regression models in order to test a potential mediation effect and to include latent variables.

**6. Harshness and unpredictability questions separated**

|  | **Harshness** |
| --- | --- |
|  | Extrinsic mortality |
| 4 | Did your mother die before the age of 18? |
| 5 | Did your father die before the age of 18? |
| 7 | From your birth until you were 7 years old, did one of your sibling die? |
|  | Morbidity |
| 13 | From your birth until you were 7 years old, did you suffer from a long disease that required hospitalization? |
|  | SES |
| 6 | How many brothers and sisters do you have? |
| 15 | "Childhood SES" scale |
|  | My family usually had enough money for things when I was growing up. |
|  | I grew up in a relatively wealthy neighborhood. |
|  | I felt relatively wealthy compared to the other kids in my school. |
|  | **Unpredictability** |
|  | Parental involvement |
| 1 | Did your parents get divorced or separated before the age of 18? |
| 2 | Have you ever lived with a stepfather before the age of 18? |
| 3 | Were you ever placed in an institution or in a foster family? |
| 14 | "Neighbourhood stability" scale |
|  | From your birth until you were 7 years old, how many times did you move? |
|  | From your birth until you were 7 years old, how many times did you change schools? |
|  | Parental investment |
| 8 | "Parental investment" scale |
|  | My parents always seemed to care about what I was doing. |
|  | My father was always there when I needed him. |
|  | My mother was always there when I needed her. |
|  | Parental predictability |
| 9 | "Parenting style" scale |
|  | When I was growing up, someone in my house was always yelling at someone else. |
|  | Some of the punishments I received when I was a child now seem too harsh to me. |
|  | I guess you could say that I wasn’t treated as well as I should have been at home. |
|  | Safety and security |
|  | Victim of psychological, sexual of psychological abuse during childhood |
| 10 | By one or several people in my family |
| 11 | By one or several people outside my family |
| 12 | "Family difficulties" index |
|  | Alcoholic |
|  | Violent |
|  | Depressed |
|  | Mental disorder |
|  | Drugs |
|  | Judicial system |
|  | Prison |

**Supplementary Table S4:** Harshness and unpredictability questions separated

**7. Structural equation model without somatic maintenance**

This additional analysis did not include the four life-history strategy indicators relating to somatic maintenance (i.e., BMI, smoking, health status and health effort). The rest of the model is unchanged. The full model is represented in Supplementary Figure S1.


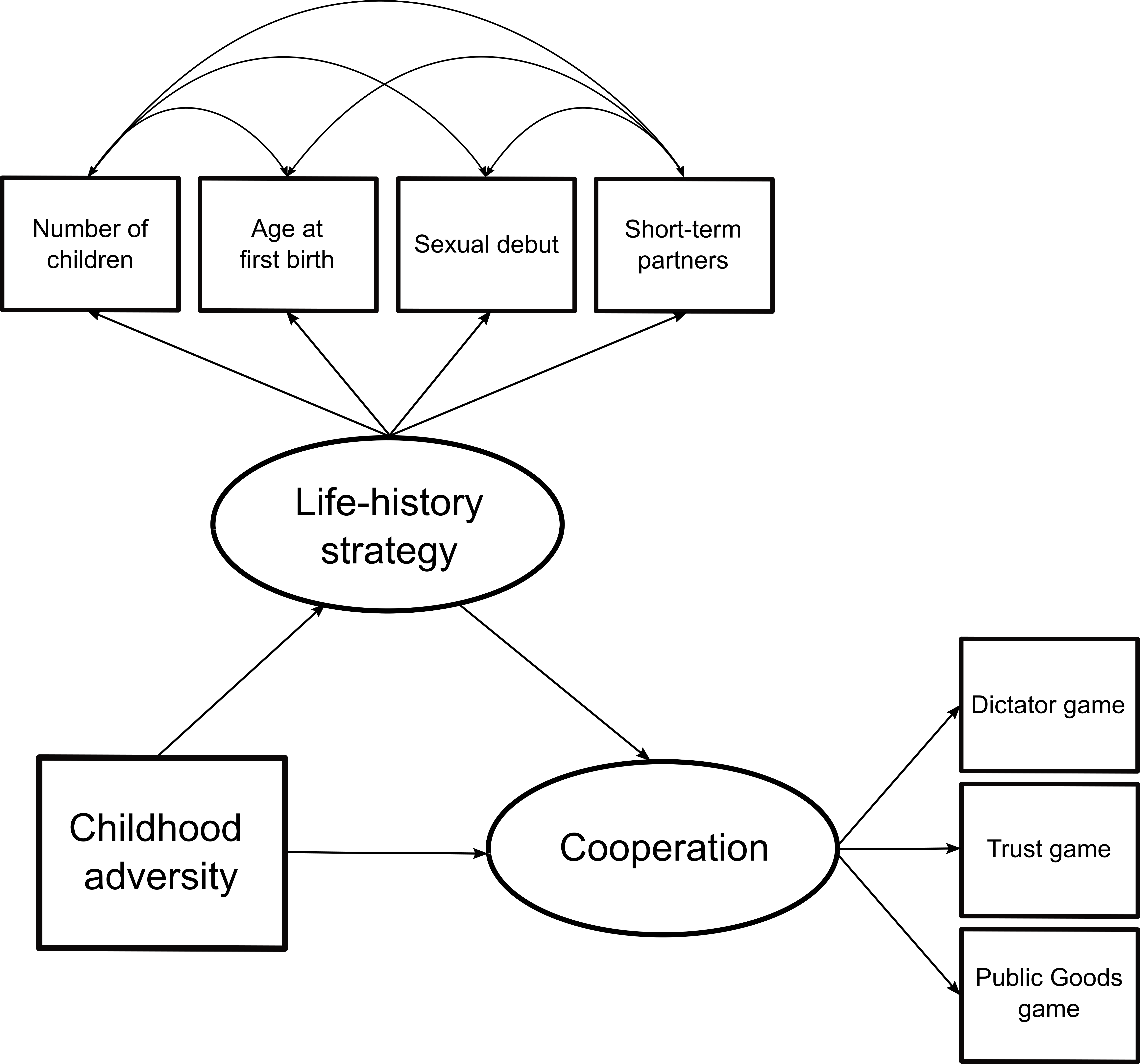


**Supplementary Figure S1:** Structural equation model without somatic maintenance

**8. Cross-validation analyses**

Cross-validation requires the split of the full dataset, typically into 5 or 10 folds, with each fold randomly composed of a training dataset (80% of the data in the case of 5 folds) and a test dataset (20% of the data in the case of 5 folds). In the present study, we chose a 5-folds cross-validation; resulting in a training dataset of 489 participants and a test dataset of 123 participants (the full dataset included 612 participants). The analyses consisted of the following steps:

1. First, the full dataset was portioned into 5 folds of nearly equal size.
2. Subsequently 5 iterations of training and validation were performed such that within each iteration a different fold of the data is held-out for validation (the test data: here representing 20% of the whole sample) while the remaining *k*-1 folds were used for fitting (the training data: here representing 80% of the whole sample).
3. At each iteration, the model-implied covariance matrix estimated from the training data was compared to the covariance matrix directly observed in the test data. Therefore, the smaller the discrepancy between the two matrices, the greater the capacity of the model to extend its predictions to out-of-sample data. The discrepancy between matrices was simply estimated by calculating the *SRMR* absolute fit index (Hu & Bentler, 1999) at each iteration, hence used as an index of the model’s cross-validation performance (reported in the present manuscript under the label *SRMR*.*cv.real*). A one-sample Wilcoxon sum of rank test was then run to investigate whether the cross-validated *SRMR* values differed from the standard acceptability cut-off of <0.10. A biserial correlation coefficient *r* was finally calculated as an index of effect size (< 0 – 0.30 = small; 0.30 – 0.50 = medium; > 0.50 = large).
4. The predictive value of the training model was further checked by applying the same 3 steps on a test dataset whose individual values were randomly permuted within each indicator. The aim was to check that the fitted values obtained when modelling the training data failed to predict the test data when its internal structure was broken down by randomization. The cross-validated *SRMR* index was also calculated (labelled *SRMR*.*cv.rand*). A two-samples Wilcoxon sum of rank test (one-tailed, because we hypothesized that the training model always predicts the test data better than the random data) was run to analyze whether the training model better predicts the real test data or the randomly permuted test data. A biserial correlation coefficient *r* was finally calculated as an index of effect size (< 0 – 0.30 = small; 0.30 – 0.50 = medium; > 0.50 = large).
5. At the end of the 5 iterations, the whole sample was reshuffled and re-stratified before a new round of iterations started.
6. The whole procedure was repeated within 200 rounds (leading to a total of 1000 iterations of the model) in order to obtain reliable performance estimation. Note that the training and test datasets always included different data points at each iteration within a round.

**9. Results main model**

| Model part | Latent variables |  | Indicators | c.unstd | se | z | p-val | ci.lower | ci.upper | c.std |
| --- | --- | --- | --- | --- | --- | --- | --- | --- | --- | --- |
| Measurement model | Life-history strategy | =~ | BMI | 0.11 | 0.06 | 2.00 | <0.05 | 0.00 | 0.22 | 0.22 |
|  |  | =~ | Smoking | 0.14 | 0.04 | 3.53 | 4.18e-04 | 0.06 | 0.22 | 0.56 |
|  |  | =~ | Health effort | -0.14 | 0.21 | -0.69 | 0.49 | -0.57 | 0.27 | -0.08 |
|  |  | =~ | Health status | -0.22 | 0.08 | -2.56 | 0.01 | -0.38 | -0.05 | -0.33 |
|  |  | =~ | Number of children | -0.06 | 0.06 | -0.91 | 0.36 | -0.19 | 0.07 | -0.07 |
|  |  | =~ | Age at 1st birth | -0.08 | 0.04 | -2.01 | 0.04 | -0.15 | 0.00 | -0.16 |
|  |  | =~ | Sexual debut | -0.10 | 0.03 | -3.76 | 1.69e-04 | -0.15 | -0.05 | -0.28 |
|  |  | =~ | Short-term partners | 0.31 | 0.17 | 1.80 | 0.07 | -0.03 | 0.64 | 0.22 |
|  | Cooperation | =~ | Dictator game | 0.82 | 0.15 | 5.33 | 9.76e-08 | 0.52 | 1.13 | 0.45 |
|  |  | =~ | Trust game | 1.88 | 0.24 | 7.78 | 7.26e-15 | 1.40 | 2.36 | 0.71 |
|  |  | =~ | Public Goods game | 1.39 | 0.20 | 6.85 | 7.37e-12 | 0.99 | 1.79 | 0.48 |
| Structural model | (a) Life-history strategy | ~ | Childhood adversity | 1.92 | 0.62 | 3.14 | <0.01 | 0.72 | 3.15 | 0.26 |
|  | (b) Cooperation | ~ | Life-history strategy | 0.12 | 0.09 | 1.30 | 0.19 | -0.06 | 0.31 | 0.12 |
|  | (c) Cooperation | ~ | Childhood adversity | -0.29 | 0.57 | -0.51 | 0.61 | -1.42 | 0.83 | -0.04 |
|  | Indirect effect | := | a * b | 0.23 | 0.22 | 1.05 | 0.29 | -0.19 | 0.67 | 0.03 |
| Covariances | BMI | ~~ | Health status | -0.04 | 0.03 | -1.58 | 0.11 | -0.09 | 0.01 | -0.13 |
|  | BMI | ~~ | Health effort | -0.12 | 0.06 | -1.98 | <0.05 | -0.24 | 0.00 | -0.13 |
|  | BMI | ~~ | Smoking | -0.01 | 0.01 | -1.12 | 0.26 | -0.04 | 0.01 | -0.13 |
|  | Smoking | ~~ | Health status | 0.02 | 0.02 | 1.29 | 0.20 | -0.01 | 0.06 | 0.18 |
|  | Smoking | ~~ | Health effort | -0.06 | 0.04 | -1.55 | 0.12 | -0.13 | 0.02 | -0.15 |
|  | Health status | ~~ | Health effort | 0.27 | 0.08 | 3.45 | 5.70e-04 | 0.12 | 0.43 | 0.23 |
|  | Number of children | ~~ | Age at 1st birth | -0.14 | 0.02 | -6.36 | 2.08e-10 | -0.19 | -0.10 | -0.33 |
|  | Number of children | ~~ | Sexual debut | 0.00 | 0.02 | -0.12 | 0.78 | -0.03 | 0.03 | -0.01 |
|  | Number of children | ~~ | Short-term partners | -0.10 | 0.07 | -1.30 | 0.19 | -0.24 | 0.05 | -0.07 |
|  | Age at 1st birth | ~~ | Sexual debut | 0.03 | 0.01 | 2.77 | <0.01 | 0.01 | 0.05 | 0.18 |
|  | Age at 1st birth | ~~ | Short-term partners | 0.10 | 0.04 | 2.42 | 0.02 | 0.02 | 0.18 | 0.14 |
|  | Sexual debut | ~~ | Short-term partners | -0.05 | 0.03 | -1.82 | 0.07 | -0.10 | 0.00 | -0.10 |

**Supplementary Table S5:** Results main model

**Cross-validation results main model**

The cross-validated *SRMR* (*SRMR.cv.real* = 0.089) differed negatively (*V* = 17023, *p* < 2.2e-16) and to a large extent (*r* = -0.796) from the standard acceptability cut-off of <0.10, suggesting that the model parameters estimated from the training data also fit the test dataset (20% of the data) satisfactorily.

In line with this result, a paired-samples Wilcoxon sum of rank test and its related effect size showed that *SRMR.cv.rand* was higher than *SRMR.cv.real* (0.131 *vs.* 0.089, *W* = 464, *p* < 2.2e-16, *r* = 0.865), i.e., the median *SRMR* extracted from the cross-validation performed on the randomly permuted test datasets. In other words, model parameters estimated from the training dataset predicted the real test data better than the random test data. One can therefore conclude from these results that the overall hypothesized latent structure of the model is present in the data.

**10. Results controlled for current environmental harshness**

| Model part | Latent variables |  | Indicators | UnStd.c | SE | z | p | ci.lower | ci.upper | Std.c |
| --- | --- | --- | --- | --- | --- | --- | --- | --- | --- | --- |
| Measurement model | Life-history strategy | =~ | BMI | 0.07 | 0.06 | 1.08 | 0.28 | -0.06 | 0.20 | 0.14 |
|  |  | =~ | Smoking | 0.13 | 0.06 | 2.39 | 0.01 | 0.03 | 0.23 | 0.52 |
|  |  | =~ | Health effort | -0.02 | 0.25 | -0.06 | 0.87 | -0.51 | 0.49 | -0.01 |
|  |  | =~ | Health status | -0.14 | 0.10 | -1.50 | 0.12 | -0.33 | 0.04 | -0.21 |
|  |  | =~ | Number of children | -0.07 | 0.08 | -0.93 | 0.32 | -0.23 | 0.08 | -0.08 |
|  |  | =~ | Age at 1st birth | -0.05 | 0.05 | -1.14 | 0.24 | -0.14 | 0.04 | -0.11 |
|  |  | =~ | Sexual debut | -0.09 | 0.04 | -2.45 | <0.01 | -0.16 | -0.02 | -0.25 |
|  |  | =~ | Short-term partners | 0.26 | 0.19 | 1.49 | 0.13 | -0.09 | 0.63 | 0.18 |
|  | Cooperation | =~ | Dictator game | 0.83 | 0.16 | 4.70 | 2.57e-06 | 0.46 | 1.19 | 0.45 |
|  |  | =~ | Trust game | 1.87 | 0.28 | 6.83 | 8.53e-12 | 1.32 | 2.43 | 0.71 |
|  |  | =~ | Public Goods game | 1.40 | 0.22 | 6.12 | 9.44e-10 | 0.93 | 1.88 | 0.49 |
| Structural model | (a) Life-history strategy | ~ | Childhood adversity | 1.37 | 0.67 | 2.06 | 0.03 | 0.07 | 2.71 | 0.19 |
|  | (b) Cooperation | ~ | Life-history strategy | 0.09 | 0.10 | 0.82 | 0.40 | -0.12 | 0.32 | 0.09 |
|  | (c) Cooperation | ~ | Childhood adversity | -0.25 | 0.61 | -0.36 | 0.71 | -1.57 | 1.04 | -0.03 |
|  | Indirect effect | := | a * b | 0.17 | 0.21 | 0.79 | 0.43 | -0.24 | 0.60 | 0.02 |
| Covariances | BMI | ~~ | Health status | -0.04 | 0.03 | -1.36 | 0.17 | -0.09 | 0.02 | -0.12 |
|  | BMI | ~~ | Health effort | -0.10 | 0.06 | -1.47 | 0.14 | -0.23 | 0.03 | -0.11 |
|  | BMI | ~~ | Smoking | -0.01 | 0.01 | -0.74 | 0.46 | -0.04 | 0.02 | -0.10 |
|  | Smoking | ~~ | Health status | 0.02 | 0.02 | 0.89 | 0.37 | -0.02 | 0.07 | 0.17 |
|  | Smoking | ~~ | Health effort | -0.07 | 0.04 | -1.60 | 0.11 | -0.16 | 0.02 | -0.19 |
|  | Health status | ~~ | Health effort | 0.24 | 0.09 | 2.82 | <0.01 | 0.07 | 0.42 | 0.21 |
|  | Number of children | ~~ | Age at 1st birth | -0.14 | 0.02 | -5.67 | 1.42e-08 | -0.19 | -0.09 | -0.33 |
|  | Number of children | ~~ | Sexual debut | -0.01 | 0.02 | -0.25 | 0.77 | -0.05 | 0.04 | -0.02 |
|  | Number of children | ~~ | Short-term partners | -0.09 | 0.08 | -0.98 | 0.33 | -0.26 | 0.08 | -0.07 |
|  | Age at 1st birth | ~~ | Sexual debut | 0.03 | 0.01 | 2.37 | 0.02 | 0.01 | 0.05 | 0.18 |
|  | Age at 1st birth | ~~ | Short-term partners | 0.10 | 0.04 | 2.02 | 0.04 | 0.00 | 0.19 | 0.14 |
|  | Sexual debut | ~~ | Short-term partners | -0.05 | 0.03 | -1.28 | 0.20 | -0.11 | 0.02 | -0.09 |

**Supplementary Table S6:** Results controlled for current environmental harshness

**Cross-validation results controlled for current environmental harshness**

The cross-validated *SRMR* (*SRMR.cv.real* = 0.092) differed negatively (*V* = 23373, *p* < 2.2e-16) and to a large extent (*r* = -0.718) from the standard acceptability cut-off of <0.10.

In support of this, the *SRMR* (*SRMR.cv.rand* = 0.133) cross-validated on the randomly permuted test datasets was much larger than the *SRMR* cross-validated on the real test datasets (Wilcoxon sum of rank tests – *SRMR.cv.rand* vs. *SRMR.cv.real*: *W* = 460, *p* < 2.2e-16, *r* = 0.864). The fact that the prediction of the training model better generalize to real test data than to randomized test data is an indication that the overall hypothesized latent structure of the model is present in the data.

**11. Results without somatic maintenance**

| Model part | Latent variables |  | Indicators | UnStd.c | SE | z | p | ci.lower | ci.upper | Std.c |
| --- | --- | --- | --- | --- | --- | --- | --- | --- | --- | --- |
| Measurement model | Life-history strategy | =~ | Number of children | 0.14 | 0.24 | 0.58 | 0.56 | -0.33 | 0.65 | 0.15 |
|  |  | =~ | Age at 1st birth | -0.23 | 0.09 | -2.61 | 0.01 | -0.41 | -0.06 | -0.50 |
|  |  | =~ | Sexual debut | -0.12 | 0.06 | -2.36 | 0.02 | -0.23 | -0.02 | -0.35 |
|  |  | =~ | Short-term partners | 0.14 | 0.38 | 0.37 | 0.71 | -0.58 | 0.93 | 0.10 |
|  | Cooperation | =~ | Dictator game | 0.82 | 0.17 | 4.59 | 4.38e-06 | 0.44 | 1.18 | 0.44 |
|  |  | =~ | Trust game | 1.93 | 0.29 | 6.87 | 6.38e-12 | 1.36 | 2.53 | 0.73 |
|  |  | =~ | Public Goods game | 1.38 | 0.22 | 6.02 | 1.75e-09 | 0.90 | 1.85 | 0.48 |
| Structural model | (a) Life-history strategy | ~ | Childhood adversity | 1.46 | 0.67 | 2.19 | 0.03 | 0.15 | 2.85 | 0.20 |
|  | (b) Cooperation | ~ | Life-history strategy | -0.05 | 0.11 | -0.41 | 0.67 | -0.30 | 0.18 | -0.05 |
|  | (c) Cooperation | ~ | Childhood adversity | 0.03 | 0.58 | 0.04 | 0.82 | -1.26 | 1.29 | 0.00 |
|  | Indirect effect | := | a * b | -0.10 | 0.20 | -0.46 | 0.64 | -0.51 | 0.31 | -0.01 |
| Covariances | Number of children | ~~ | Age at 1st birth | -0.09 | 0.07 | -1.26 | 0.21 | -0.24 | 0.05 | -0.26 |
|  | Number of children | ~~ | Sexual debut | 0.03 | 0.04 | 0.75 | 0.45 | -0.05 | 0.10 | 0.10 |
|  | Number of children | ~~ | Short-term partners | -0.14 | 0.13 | -1.11 | 0.27 | -0.39 | 0.10 | -0.11 |
|  | Age at 1st birth | ~~ | Short-term partners | 0.12 | 0.11 | 1.04 | 0.30 | -0.10 | 0.34 | 0.21 |
|  | Sexual debut | ~~ | Short-term partners | -0.06 | 0.06 | -0.92 | 0.36 | -0.17 | 0.06 | -0.12 |

**Supplementary Table S7:** without somatic maintenance. One of the covariances had to be removed in order for the model to converge. We chose the covariance between “Age at first child’s birth” and “Sexual debut”.

**Cross-validation results without somatic maintenance**

Once again, the *SRMR* (*SRMR.cv.real* = 0.083) differed negatively (*V* = 10098, *p* < 2.2e-16) and to a large extent (*r* = -0.827) from the standard acceptability cut-off of <0.10.

Additionally, the *SRMR* values (*SRMR.cv.rand* = 0.138) extracted from the randomly permuted test datasets was much larger than the one extracted from the real test datasets (*SRMR.cv.rand* vs. *SRMR.cv.real*: *W* = 716, *p* < 2.2e-16, *r* = 0.864). This means that the model parameters based on the training dataset better predict the real test dataset than the randomly permuted test dataset.

**12. Results harshness and unpredictability**

**Measurement model**

The standardized regression weights can be found in Supplementary Figure S2 (the full results can be found below in Supplementary Table S8).

“Dictator game” (*UnStd.c* = 0.83, *z* = 5.41, *p* = 6.39e-08, *Std.c* = 0.45), “Trust game” (*UnStd.c* = 1.87, *z* = 7.88, *p* = 3.18e-15, *Std.c* = 0.71) and “Public Goods game” (*UnStd.c* = 1.39, *z* = 6.94, *p* = 3.89e-012, *Std.c* = 0.49) loaded significantly on the cooperation latent variable, whose greater values indicate higher initial contributions in the economic games. The average explained variance (R^2^) of the individual indicators by the cooperation latent variable is 0.32, which is considered substantial (Cohen, 1988).

Five out of the eight indicators loaded significantly on the life-history strategy latent variable: “BMI” (*UnStd.c* = 0.11, *z* = 2.07, *p* = 0.04, *Std.c* = 0.22), “Smoking” (*UnStd.c* = 0.13, *z* = 3.91, *p* = 9.24e-05, *Std.c* = 0.53), “Health status” (*UnStd.c* = -0.21, *z* = -2.75, *p* < 0.01, *Std.c* = -0.31), “Age at first child’s birth” (*UnStd.c* = -0.08, *z* = -2.06, *p* = 0.04, *Std.c* = -0.17) and “Sexual debut” (*UnStd.c* = -0.10, *z* = -4.08, *p* = 4.60e-05, *Std.c* = -0.30). “Short-term partners” marginally correlated with scores on the life-history strategy latent variable (*UnStd.c* = 0.30, *z* = 1.79, *p* = 0.07, *Std.c* = 0.21). “Health effort” (*p* = 0.51) and “Number of children” (*p* = 0.34) were not captured by the life-history strategy latent variable. The average explained variance (R^2^) of the significant indicators by the life-history strategy latent variable is 0.14, which is considered moderate (Cohen, 1988).

Estimated covariances revealed correlations between most somatic maintenance indicators. Specifically, “BMI” and “Health effort” (*UnStd.c* = -0.12, *z* = -2.09, *p* = 0.04, *Std.c* = -0.13) were negatively associated. “Health status” and “Health effort” (*UnStd.c* = 0.28, *z* = 3.69, *p* = 2.26e-04, *Std.c* = 0.23) were positively associated. “BMI” and “Health status” (*UnStd.c* = -0.04, *z* = -1.78, *p* = 0.07, *Std.c* = -0.13) were negatively and marginally correlated. “Smoking” and “Health effort” (*UnStd.c* = -0.06, *z* = -1.81, *p* = 0.07, *Std.c* = -0.15) were negatively and marginally correlated.

Estimated covariances revealed correlations between most of the reproduction indicators. Specifically, “Number of children” and “Age at first child’s birth” (*UnStd.c* = -0.14, *z* = -6.33, *p* = 2.49e-10, *Std.c* = -0.33) were negatively associated. “Age at first child’s birth” and “Sexual debut” (*UnStd.c* = 0.03, *z* = 2.75, *p* < 0.01, *Std.c* = 0.18) were positively associated. “Age at first child’s birth” and “Short-term partners” (*UnStd.c* = 0.10, *z* = 2.47, *p* = 0.01, *Std.c* = 0.15) were positively associated. “Sexual debut” and “Short-term partners” (*UnStd.c* = -0.05, *z* = -1.78, *p* = 0.08, *Std.c* = -0.10) were negatively and marginally correlated.


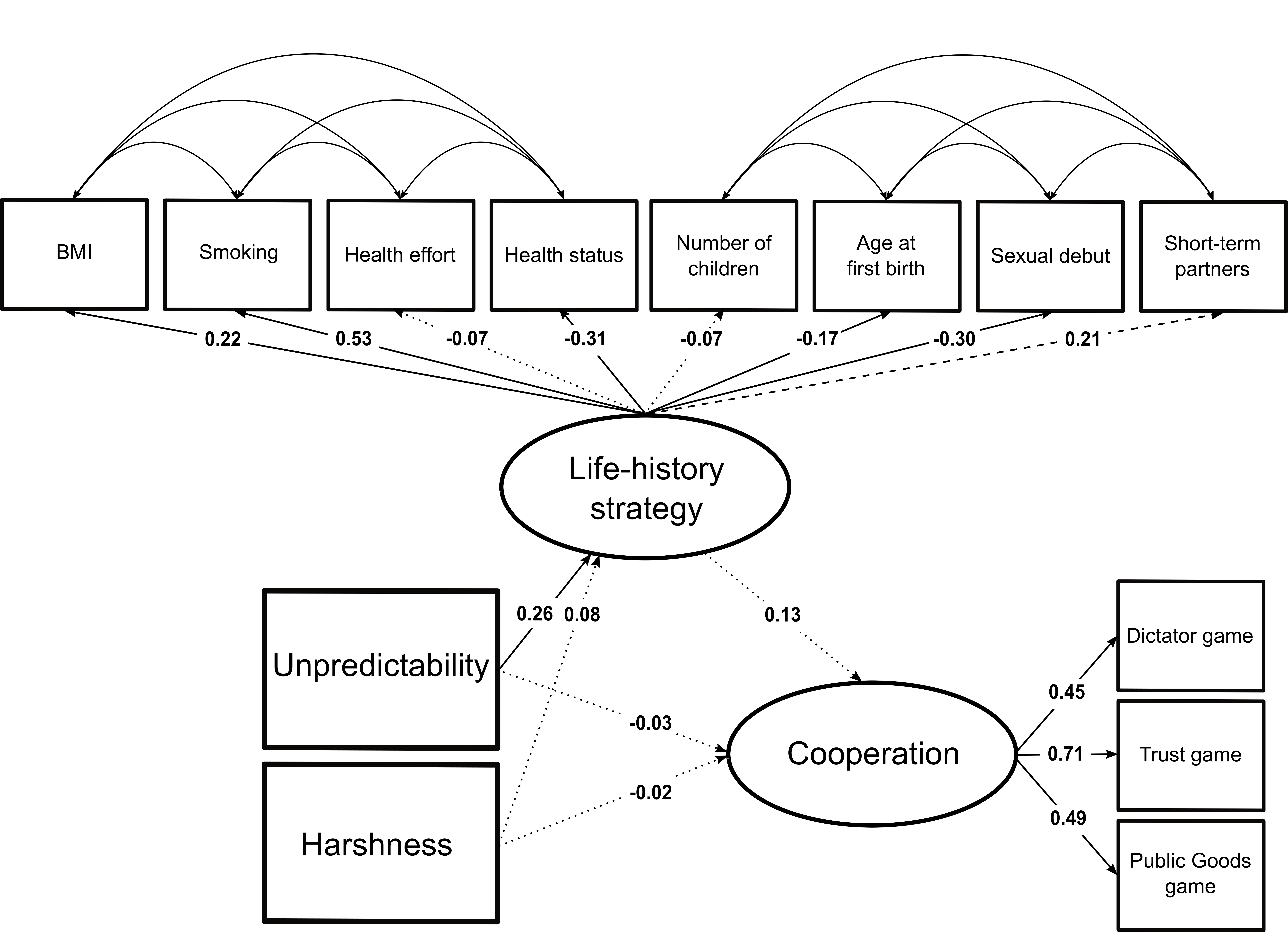


**Supplementary Figure S2:** Standardized parameter values estimated by the structural equation model. Significant paths at the 5% level are represented with a continuous arrow, marginally significant paths at the 10% level are represented with a dashed arrow and non-significant paths are represented with a dotted arrow.

**Structural model**

Supplementary Figure S2 shows that neither childhood unpredictability (*UnStd.c* = -0.03, *z* = -0.44, *p* = 0.64, *Std.c* = -0.03) nor childhood harshness (*UnStd.c* = -0.02, *z* = -0.32, *p* = 0.72, *Std.c* = -0.02) are associated with cooperation. However, childhood unpredictability is significantly associated with life-history strategy (*UnStd.c* = 0.21, *z* = 3.26, *p* < 0.01, *Std.c* = 0.26). Overall, a more unpredictable childhood environment is associated with an increased investment in reproduction and a decreased investment in somatic maintenance. Childhood harshness is not associated with life-history strategy (*UnStd.c* = 0.05, *z* = 1.07, *p* = 0.29, *Std.c* = 0.08). Overall, the explained variance (R^2^) of the life-history strategy latent variable is 0.08, which is considered weak to moderate (Cohen, 1988). Finally, life-history strategy is not significantly associated with cooperation (*UnStd.c* = 0.12, *z* = 1.32, *p* = 0.19, *Std.c* = 0.13). The explained variance (R^2^) of the cooperation latent variable is 0.02, which is considered weak (Cohen, 1988).

Consequently, the indirect effect of childhood unpredictability on cooperation via life-history strategy is not significant (indirect effect with the Delta method: *UnStd.c* = 0.02, *z* = 1.10, *p* = 0.27, *Std.c* = 0.03). A null result is also found with the indirect effect of childhood harshness on cooperation via life-history strategy (indirect effect with the Delta method: *UnStd.c* = 0.01, *z* = 0.67, *p* = 0.50, *Std.c* = 0.01).

| Model part | Latent variables |  | Indicators | UnStd.c | SE | z | p | ci.lower | ci.upper | Std.c |
| --- | --- | --- | --- | --- | --- | --- | --- | --- | --- | --- |
| Measurement model | Life-history strategy | =~ | BMI | 0.11 | 0.05 | 2.07 | 0.04 | 0.01 | 0.21 | 0.22 |
|  |  | =~ | Smoking | 0.13 | 0.03 | 3.91 | 9.24e-05 | 0.07 | 0.20 | 0.53 |
|  |  | =~ | Health effort | -0.13 | 0.20 | -0.66 | 0.51 | -0.51 | 0.25 | -0.07 |
|  |  | =~ | Health status | -0.21 | 0.07 | -2.75 | <0.01 | -0.36 | -0.06 | -0.31 |
|  |  | =~ | Number of children | -0.06 | 0.07 | -0.96 | 0.34 | -0.19 | 0.07 | -0.07 |
|  |  | =~ | Age at 1st birth | -0.08 | 0.04 | -2.06 | 0.04 | -0.15 | 0.00 | -0.17 |
|  |  | =~ | Sexual debut | -0.10 | 0.03 | -4.08 | 4.60e-05 | -0.15 | -0.05 | -0.30 |
|  |  | =~ | Short-term partners | 0.30 | 0.17 | 1.79 | 0.07 | -0.03 | 0.64 | 0.21 |
|  | Cooperation | =~ | Dictator game | 0.83 | 0.15 | 5.41 | 6.39e-08 | 0.53 | 1.13 | 0.45 |
|  |  | =~ | Trust game | 1.87 | 0.24 | 7.88 | 3.18e-15 | 1.41 | 2.34 | 0.71 |
|  |  | =~ | Public Goods game | 1.39 | 0.20 | 6.94 | 3.89e-12 | 1.00 | 1.78 | 0.49 |
| Structural model | (a1) Life-history strategy | ~ | Unpredictability | 0.21 | 0.06 | 3.26 | <0.01 | 0.08 | 0.34 | 0.26 |
|  | (a2) Life-history strategy | ~ | Harshness | 0.05 | 0.05 | 1.07 | 0.29 | -0.04 | 0.15 | 0.08 |
|  | (b) Cooperation | ~ | Life-history strategy | 0.12 | 0.09 | 1.32 | 0.19 | -0.06 | 0.30 | 0.13 |
|  | (c1) Cooperation | ~ | Unpredictability | -0.03 | 0.06 | -0.44 | 0.64 | -0.14 | 0.09 | -0.03 |
|  | (c2) Cooperation | ~ | Harshness | -0.02 | 0.05 | -0.32 | 0.72 | -0.12 | 0.08 | -0.02 |
|  | Indirect effect | := | a1 * b | 0.02 | 0.02 | 1.10 | 0.27 | -0.02 | 0.07 | 0.03 |
|  | Indirect effect | := | a2 * b | 0.01 | 0.01 | 0.67 | 0.50 | -0.01 | 0.03 | 0.01 |
| Covariances | BMI | ~~ | Health status | -0.04 | 0.02 | -1.78 | 0.07 | -0.09 | 0.00 | -0.13 |
|  | BMI | ~~ | Health effort | -0.12 | 0.06 | -2.09 | 0.04 | -0.24 | -0.01 | -0.13 |
|  | BMI | ~~ | Smoking | -0.01 | 0.01 | -1.14 | 0.25 | -0.03 | 0.01 | -0.11 |
|  | Smoking | ~~ | Health status | 0.02 | 0.02 | 1.36 | 0.17 | -0.01 | 0.05 | 0.15 |
|  | Smoking | ~~ | Health effort | -0.06 | 0.03 | -1.81 | 0.07 | -0.13 | 0.00 | -0.15 |
|  | Health status | ~~ | Health effort | 0.28 | 0.08 | 3.69 | 2.26e-04 | 0.13 | 0.43 | 0.23 |
|  | Number of children | ~~ | Age at 1st birth | -0.14 | 0.02 | -6.33 | 2.49e-10 | -0.18 | -0.10 | -0.33 |
|  | Number of children | ~~ | Sexual debut | 0.00 | 0.02 | -0.22 | 0.75 | -0.04 | 0.03 | -0.01 |
|  | Number of children | ~~ | Short-term partners | -0.10 | 0.07 | -1.33 | 0.18 | -0.24 | 0.05 | -0.07 |
|  | Age at 1st birth | ~~ | Sexual debut | 0.03 | 0.01 | 2.75 | <0.01 | 0.01 | 0.05 | 0.18 |
|  | Age at 1st birth | ~~ | Short-term partners | 0.10 | 0.04 | 2.47 | 0.01 | 0.02 | 0.18 | 0.15 |
|  | Sexual debut | ~~ | Short-term partners | -0.05 | 0.03 | -1.78 | 0.08 | -0.10 | 0.00 | -0.10 |

**Supplementary Table S8:** Results harshness and unpredictability

**Cross-validation results harshness and unpredictability**

The cross-validated *SRMR* values of this model (*SRMR.cv.real* = 0.090) differed negatively (*V* = 18538, *p* < 0.001) and to a large extent (*r* = -0.799) from the standard cut-off of <0.10.

The capacity of the harshness and unpredictability model to predict random test data was poorer in comparison to the real test data: the *SRMR* values (*SRMR.cv.rand* = 0.128) were indeed much larger (*SRMR.cv.rand* vs. *SRMR.cv.real*: *W* = 339, *p* < 0.001, *r* = 0.865). The model parameters estimated from the training data therefore predicted the real test data better than the random test data.

**13. Results self-reported social trust**

| Model part | Latent variables |  | Indicators | UnStd.c | SE | z | p | ci.lower | ci.upper | Std.c |
| --- | --- | --- | --- | --- | --- | --- | --- | --- | --- | --- |
| Measurement model | Life-history strategy | =~ | BMI | 0.11 | 0.06 | 1.92 | 0.06 | 0.00 | 0.22 | 0.22 |
|  |  | =~ | Smoking | 0.13 | 0.04 | 3.58 | 3.42e-04 | 0.06 | 0.20 | 0.54 |
|  |  | =~ | Health effort | -0.16 | 0.21 | -0.80 | 0.42 | -0.57 | 0.24 | -0.09 |
|  |  | =~ | Health status | -0.19 | 0.08 | -2.37 | 0.02 | -0.35 | -0.03 | -0.29 |
|  |  | =~ | Number of children | -0.05 | 0.07 | -0.67 | 0.50 | -0.19 | 0.09 | -0.05 |
|  |  | =~ | Age at 1st birth | -0.09 | 0.04 | -2.23 | 0.03 | -0.17 | -0.01 | -0.19 |
|  |  | =~ | Sexual debut | -0.10 | 0.03 | -3.82 | 1.33e-04 | -0.15 | -0.05 | -0.29 |
|  |  | =~ | Short-term partners | 0.30 | 0.18 | 1.72 | 0.09 | -0.04 | 0.65 | 0.21 |
| Structural model | (a) Life-history strategy | ~ | Childhood adversity | 2.08 | 0.64 | 3.25 | <0.01 | 0.83 | 3.35 | 0.28 |
|  | (b) Self-reported social trust | ~ | Life-history strategy | -0.05 | 0.08 | -0.72 | 0.47 | -0.21 | 0.10 | -0.06 |
|  | (c) Self-reported social trust | ~ | Childhood adversity | -0.11 | 0.43 | -0.24 | 0.75 | -0.96 | 0.75 | -0.02 |
|  | Indirect effect | := | a * b | -0.11 | 0.19 | -0.59 | 0.55 | -0.47 | 0.25 | -0.02 |
| Covariances | BMI | ~~ | Health status | -0.05 | 0.02 | -1.85 | 0.06 | -0.09 | 0.00 | -0.14 |
|  | BMI | ~~ | Health effort | -0.12 | 0.06 | -1.99 | <0.05 | -0.24 | 0.00 | -0.13 |
|  | BMI | ~~ | Smoking | -0.01 | 0.01 | -1.01 | 0.31 | -0.03 | 0.01 | -0.11 |
|  | Smoking | ~~ | Health status | 0.02 | 0.02 | 1.10 | 0.27 | -0.01 | 0.05 | 0.13 |
|  | Smoking | ~~ | Health effort | -0.05 | 0.04 | -1.41 | 0.16 | -0.13 | 0.02 | -0.13 |
|  | Health status | ~~ | Health effort | 0.27 | 0.08 | 3.39 | 7.07e-04 | 0.11 | 0.43 | 0.22 |
|  | Number of children | ~~ | Age at 1st birth | -0.14 | 0.02 | -6.10 | 1.07e-09 | -0.18 | -0.09 | -0.33 |
|  | Number of children | ~~ | Sexual debut | 0.00 | 0.02 | -0.01 | 0.79 | -0.03 | 0.03 | 0.00 |
|  | Number of children | ~~ | Short-term partners | -0.10 | 0.07 | -1.37 | 0.17 | -0.25 | 0.04 | -0.08 |
|  | Age at 1st birth | ~~ | Sexual debut | 0.03 | 0.01 | 2.54 | 0.01 | 0.01 | 0.05 | 0.17 |
|  | Age at 1st birth | ~~ | Short-term partners | 0.10 | 0.04 | 2.37 | 0.02 | 0.02 | 0.19 | 0.15 |
|  | Sexual debut | ~~ | Short-term partners | -0.05 | 0.03 | -1.71 | 0.09 | -0.10 | 0.01 | -0.10 |

**Supplementary Table S9:** Results self-reported social trust

**Cross-validation results self-reported social trust**

A one-sample Wilcoxon sum of rank test showed that the cross-validated *SRMR* (*SRMR.cv.real* = 0.091) stayed below the edge of acceptability (*V* = 14862, *p* < 0.001, *r* = -0.708).

The capacity of the model to predict random test data was again poorer in comparison to the real test data: the *SRMR* values (*SRMR.cv.rand* = 0.132) were indeed much larger (*SRMR.cv.rand* vs. *SRMR.cv.real*: W = 684, *p* < 0.001, *r* = 0.862). Again, the model parameters estimated from the training data therefore predicted the real test data better than the random test data.

**References**

Cohen, J. (1988). *Statistical Power Analysis for the Behavioral Sciences*. Hillsdale, NJ: Laurence Erlbaum Associates.

Hu, L. T., & Bentler, P. M. (1999). Cutoff criteria for fit indexes in covariance structure analysis: Conventional criteria versus new alternatives. *Structural Equation Modeling: A Multidisciplinary Journal*, *6*(1), 1–55. https://doi.org/10.1080/10705519909540118

Lettinga, N., Jacquet, P. O., André, J.-B., Baumard, N., & Chevallier, C. (2020). Environmental adversity is associated with lower investment in collective actions. *PloS One*, *15*(7), e0236715. https://doi.org/10.1371/journal.pone.0236715

Mell, H., Safra, L., Algan, Y., Baumard, N., & Chevallier, C. (2018). Childhood environmental harshness predicts coordinated health and reproductive strategies: A cross-sectional study of a nationally representative sample from France. *Evolution and Human Behavior*, *39*(1), 1–8. https://doi.org/10.1016/j.evolhumbehav.2017.08.006
